# A multi-omics approach for biomarker discovery in neuroblastoma: a network-based framework

**DOI:** 10.1038/s41540-024-00371-3

**Published:** 2024-05-17

**Authors:** Rahma Hussein, Ahmed M. Abou-Shanab, Eman Badr

**Affiliations:** 1 Biomedical Sciences Program, University of Science and Technology, Zewail City of Science and Technology, Giza, 12578 Egypt; 2 Faculty of Computers and Artificial Intelligence, Cairo University, Giza, 12613 Egypt

**Keywords:** Computational biology and bioinformatics, Cancer, Biomarkers

## Abstract

Neuroblastoma (NB) is one of the leading causes of cancer-associated death in children. MYCN amplification is a prominent genetic marker for NB, and its targeting to halt NB progression is difficult to achieve. Therefore, an in-depth understanding of the molecular interactome of NB is needed to improve treatment outcomes. Analysis of NB multi-omics unravels valuable insight into the interplay between MYCN transcriptional and miRNA post-transcriptional modulation. Moreover, it aids in the identification of various miRNAs that participate in NB development and progression. This study proposes an integrated computational framework with three levels of high-throughput NB data (mRNA-seq, miRNA-seq, and methylation array). Similarity Network Fusion (SNF) and ranked SNF methods were utilized to identify essential genes and miRNAs. The specified genes included both miRNA-target genes and transcription factors (TFs). The interactions between TFs and miRNAs and between miRNAs and their target genes were retrieved where a regulatory network was developed. Finally, an interaction network-based analysis was performed to identify candidate biomarkers. The candidate biomarkers were further analyzed for their potential use in prognosis and diagnosis. The candidate biomarkers included three TFs and seven miRNAs. Four biomarkers have been previously studied and tested in NB, while the remaining identified biomarkers have known roles in other types of cancer. Although the specific molecular role is yet to be addressed, most identified biomarkers possess evidence of involvement in NB tumorigenesis. Analyzing cellular interactome to identify potential biomarkers is a promising approach that can contribute to optimizing efficient therapeutic regimens to target NB vulnerabilities.

## Introduction

Neuroblastoma (NB) is one of the most common solid malignant cancers in new births and has a sympathoadrenal origin^1,2^. NB patients are mostly diagnosed before age 10, and the age at which diagnosis occurs is inversely related to treatment outcomes^3,4^. Distinguishable from other pediatric tumors, infants with low-risk tumors have an 88% survival rate, which has been improved through advances in the cancer biology field. Moreover, NB encounters spontaneous regression or differentiates into benign ganglioneuromas^5,6^. Contrarily, the survival rate for older children, specifically those between 18 months and 12 years, is ~49%. However, in young adults (above 12 years), the survival rate plunges below 10% due to the predominance of extensive hematogenous metastasis as NB progresses^7^. Hence, this increases death cases despite the continuous multimodal therapeutics^8,9^. The clinical treatment and presentation of high-risk NB bring on relapse and a refractory state after significant responses to the initial chemotherapy.

MYCN gene amplification is the best-known prognostic marker of NB^10^. It has been established to antagonize several oncosuppressive mRNAs, like p53^11^, and miRNAs, such as miR-184^12^, indicating that MYCN exerts both transcriptional and post-transcriptional interaction on its targets. Although MYCN is the most crucial target for NB therapy, its direct targeting is challenging because of its pleiotropic effect and the difficulty of blocking transcription factors^13^. As an alternative approach, drugs could be designed to inactivate MYCN partners or transcriptional targets^14^. Consequently, the construction of a comprehensive MYCN regulatory network of various regulatory interaction types is needed. Overall, further investigations on the whole NB cell interactome orchestration are required to unravel candidate biomarkers that could be targeted to prevent NB tumorigenesis.

High-throughput technologies have enabled the global analysis of biological molecules, yielding vast amounts of omics data yearly^15^. Having multi-omics datasets for the same cohort of patients provides insight into biological interactions at different levels. It can lead to a better understanding of the molecular mechanism network that primes disease development, such as cancers. A characteristic of high-throughput multi-omics data is high dimensionality. For instance, a typical RNA-seq experiment may produce expression data for tens of thousands of transcripts. Machine learning methods help identify essential features in high-dimensional datasets and predict potential disease biomarkers. Biomarker identification contributes to understanding the underlying biology of the disease, such as identifying associated driver genes or finding potential drug targets^16^.

Several studies have utilized machine learning techniques for studying NB. A deep neural network used NB gene expression data to classify patients according to their International Neuroblastoma Staging System (INSS) stage^17^. A Concatenated Diagnostic-Relapse Prognostic deep learning model was used for NB survival prediction. The model consisted of an autoencoder coupled with a multi-task classifier. It was trained on NB transcription data to predict the Overall Survival (OS) and Event-Free Survival (EFS)^18^. In^19^, copy number alteration data was incorporated with expression data. A deep learning autoencoder combined with k-means clustering was utilized. The model identified two high-risk NB subtypes, each showing distinct survival outcomes. The authors in ref. ^20^ developed an integrative network fusion for end-point prediction of NB. It was used to analyze expression data and copy number data. Few reports tackled integrative omics approaches in identifying effectors of MYCN, critical regulators, and potential therapeutic targets in the NB^21,22^. Similarity network fusion (SNF) was used to integrate NB multi-omics data. A deep neural network and recursive feature elimination were then used on the combined data to predict patient survival. SNF achieved better integration for multi-omics data than feature-level fusion. It also showed proficiency in managing data heterogeneity and high dimensionality^23^. In another NB multi-omics study, epigenomic profiling using hmTOP-seq and uTOP-seq was performed. Transcriptomic profiling was conducted using mRNA-seq. Integrative epigenomic and transcriptomic data analysis was used to discover different cell signatures attributed to other NB cell subpopulations^24^. An NB metastasis signature consisting of 18 genes was identified through analysis of NB multi-omics. RNA-seq and copy number variation data for primary and recurrent tumors were compared. Survival analysis was utilized to identify prognostic biomarker genes^25^.

Here, we aim to identify NB biomarkers utilizing a multi-omics data integrative approach. Data describing NB’s molecular nature at the DNA methylation, mRNA, and miRNA levels were analyzed. Ranked SNF was utilized to select essential genes and miRNAs relevant to NB. Finally, network analysis was employed to identify their interaction and suggest potential biomarkers. The identified candidate biomarkers were further analyzed for their potential use as prognostic and diagnostic markers.

## Results

### Overview of the proposed framework

**Fig. 1:**
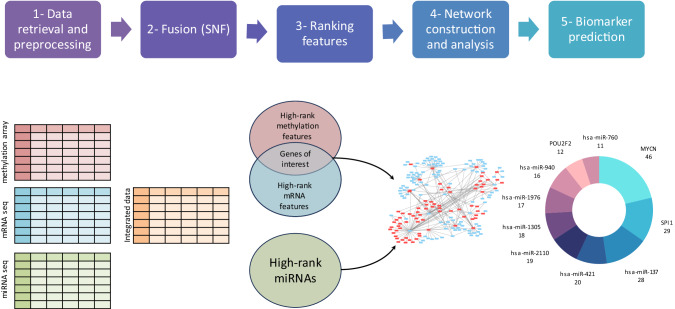
The proposed workflow. A block diagram for predicting NB candidate biomarkers.

Multi-omics data were obtained for 99 patients. The data types included were mRNA-seq, miRNA-seq, and methylation array data. Each data type was utilized to construct a patient similarity matrix. The three similarity matrices were then integrated using the SNF technique, producing a single fused similarity matrix. The Ranked Similarity Network Fusion method (rSNF) was then used to rank the features of each data type. The top 10% of high-rank features from all data types were first filtered to determine candidate biomarkers. Then, the common genes from methylation and mRNA-seq data were considered essential genes. Next, the interactions between the identified set of essential genes and the previously determined high-rank miRNAs were explored by building an interaction network. To create the network, TF-miRNA and miRNA-target interactions between the identified genes and the filtered miRNAs were retrieved from public experimental databases and integrated to construct a regulatory network. Finally, the maximal clique centrality (MCC) was performed to identify hub nodes as potential biomarkers (Fig. 1).

### Parameter tuning and data integration

**Fig. 2:**
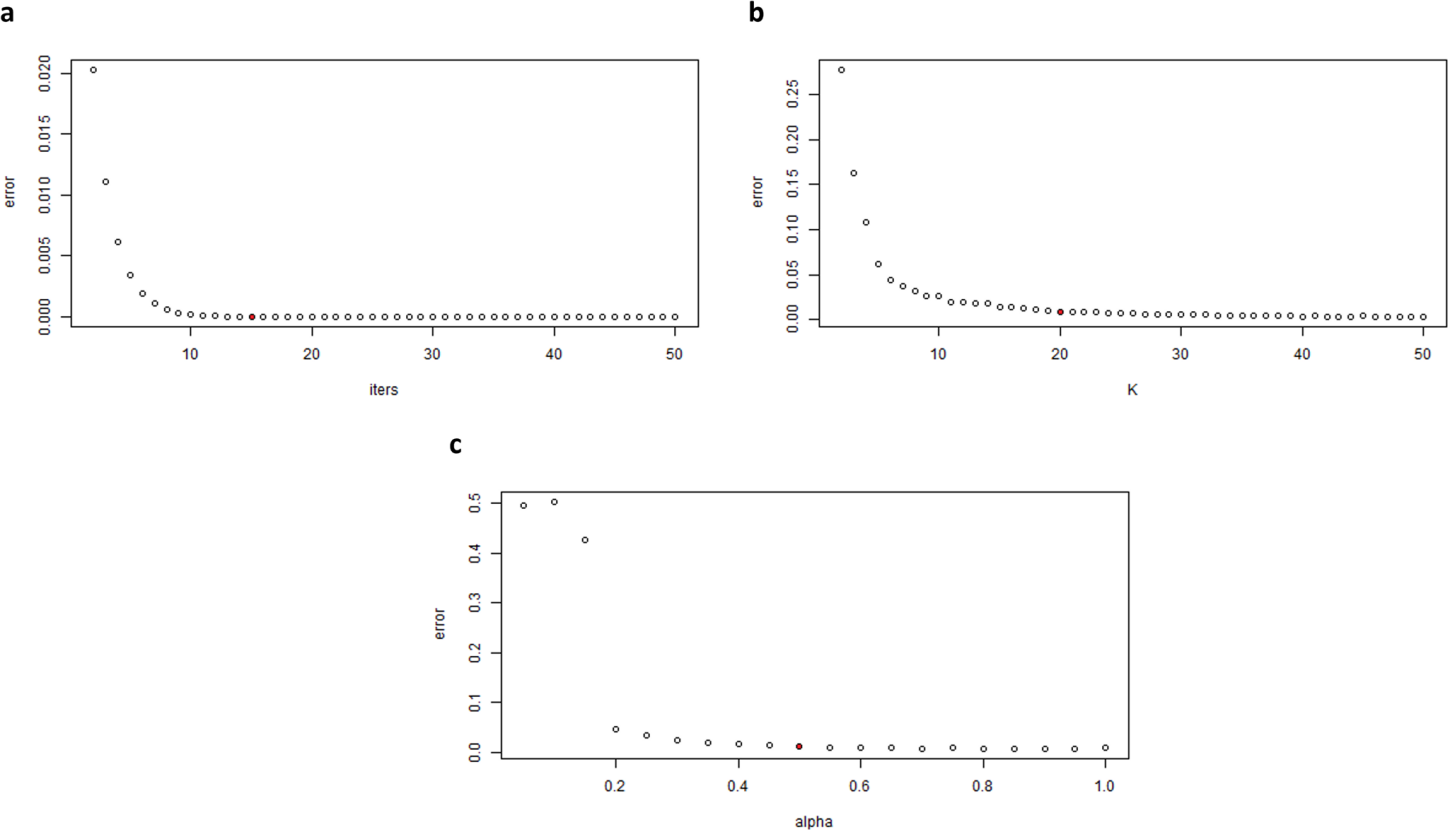
Convergence of SNF using different hyperparameters. The selected values sufficient for convergence are highlighted in red. **a** Number of iterations (*T*). **b** Number of nearest neighbors (*k*). **c** The alpha (*α*) hyperparameter (used in calculating the similarity matrix from the distance matrix). *T* = 15, *k* = 20, and *α* = 0.5 were sufficient for convergence.

**Fig. 3:**
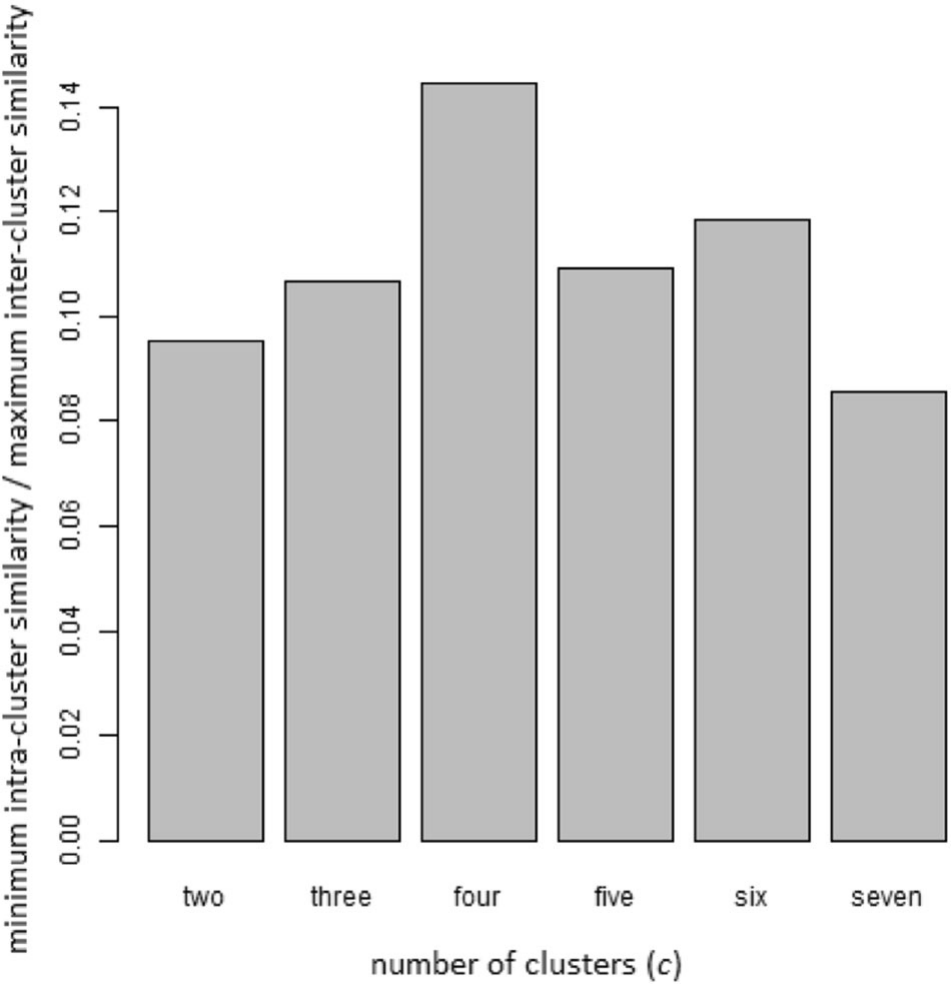
Evaluating clustering. The vertical axis corresponds to the ratio between minimum intra-cluster similarity and the maximum inter-cluster similarity. The horizontal axis shows the number of clusters used.

Different values have been evaluated iteratively for SNF hyperparameters (*T, k*, *α*) to choose the best values for SNF convergence. Figure 2 illustrates the relative change between fused graphs obtained in consecutive iterations. For the *T* parameter, each iteration is present using one more fusion iteration step where (*T*_i+1_ = *T*_i_ + 1), as shown in Fig. 2 (a,b), shows the effect of utilizing an increasing set of nearest neighbors (*k*_i+1_ = *k*_i_ + 1). Different values of *α* have also been evaluated (Fig. 2c). As shown in Fig. 2, *T* = 15, *k* = 20, and *α* = 0.5 were sufficient for convergence. Spectral clustering was performed on the fused graph using 2:7 clusters. The number of clusters was set to *c* = 4, as Fig. 3 shows, which resulted in the highest ratio between minimum intra-cluster similarity and maximum inter-cluster similarity.

### Feature selection

**Fig. 4:**
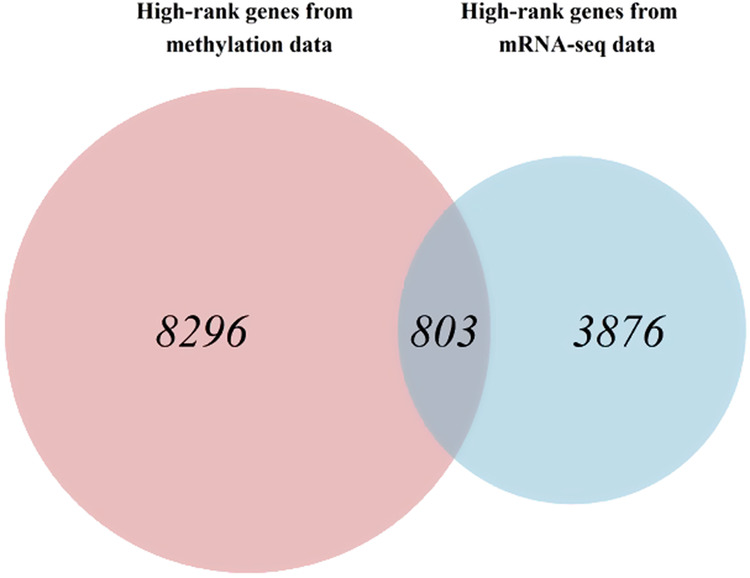
Identified essential genes. Shared genes between highly ranked genes from the methylation array and mRNA-seq data.

SNF was used to integrate the three similarity matrices into one fused similarity matrix. Then, rSNF was used to assign ranks to all features. The features were ordered by rank for each data type, and the top 10% of the features were selected. As a result, 37,953 high-rank CpG sites from the methylation array data, of which 67.8% mapped to 9099 genes, 4679 unique high-rank genes from the mRNA-seq data, and 160 high-rank miRNAs from the miRNA-seq data were selected. On comparing high-rank genes from the methylation array and mRNA-seq data, 803 genes were shared between both groups identified as essential genes (Fig. 4).

### Regulatory network construction

TF-miRNA and miRNA-target interactions that involve a high-rank miRNA and essential genes were retrieved from the transmir 2.0 and Tarbase v8 databases. The genes encoding for transcription factors were identified from the transmir 2.0 database Field, where 255 unique TF-miRNA interactions were obtained, and 161 unique miRNA-target interactions were retrieved from Tarbase v8^26^.

**Fig. 5:**
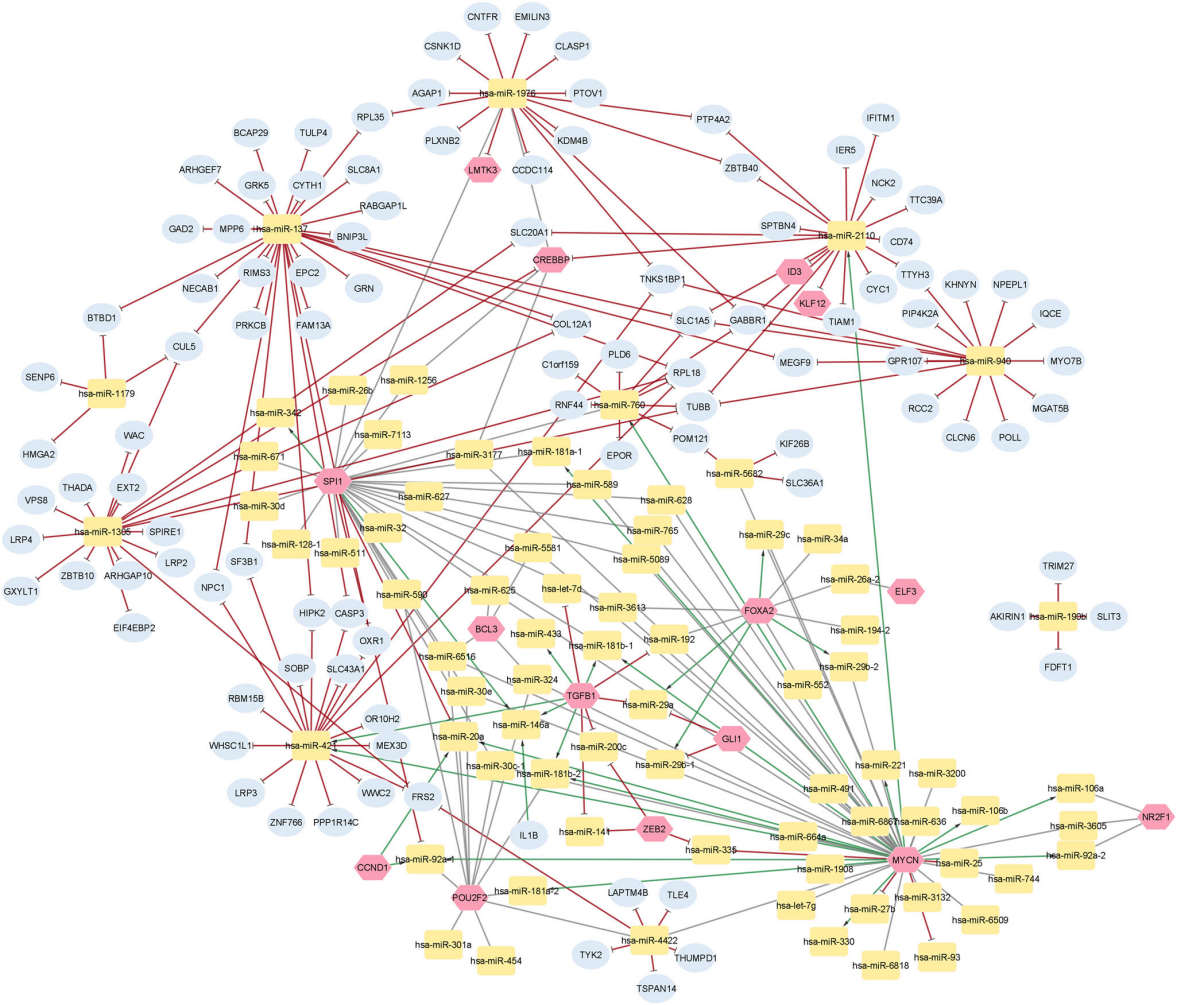
TFs-gene-miRNAs regulatory network; consists of 195 nodes and 265 edges. Node shape and color are labeled as pink hexagons representing TFs, blue ellipses representing genes, and yellow round squares representing miRNAs. The nodes have directed and colored edges: the delta-shaped green arrows indicate upregulation, while the T-shaped red arrows indicate downregulation. Black-colored edges indicate regulation.

Integrating all the interactions to construct a regulatory network resulted in 90 miRNAs, 23 TFs, and 199 target genes. The network is then visualized using Cytoscape^27^ (Fig. 5). Utilizing the MCC algorithm^28^, the top 10 hub nodes were identified and ranked, as shown in Table 1. They included three transcription factors and seven miRNAs in the regulatory network and were considered potential biomarkers for targeting NB. MYCN gene is shown to be the highest-ranked node. All the identified candidate biomarkers are implicated in NB, such as MYCN or other tumors, including breast cancers and glioma. The interaction network of the top 10 hub nodes, ranked according to the MCC score, is provided (Supplementary Fig. 1).

**Table 1:** MCC score-based ranking of the hub 10 nodes in the regulatory network

Node	Type	MCC score
MYCN	TF	46
SPI1	TF	29
Hsa-miR-137	miRNA	28
Hsa-miR-421	miRNA	20
Hsa-miR-2110	miRNA	19
Hsa-miR-1305	miRNA	18
Hsa-miR-1976	miRNA	17
Hsa-miR-940	miRNA	16
POU2F2	TF	12
Hsa-miR-760	miRNA	11

### Validation of the identified potential biomarkers

**Fig. 6:**
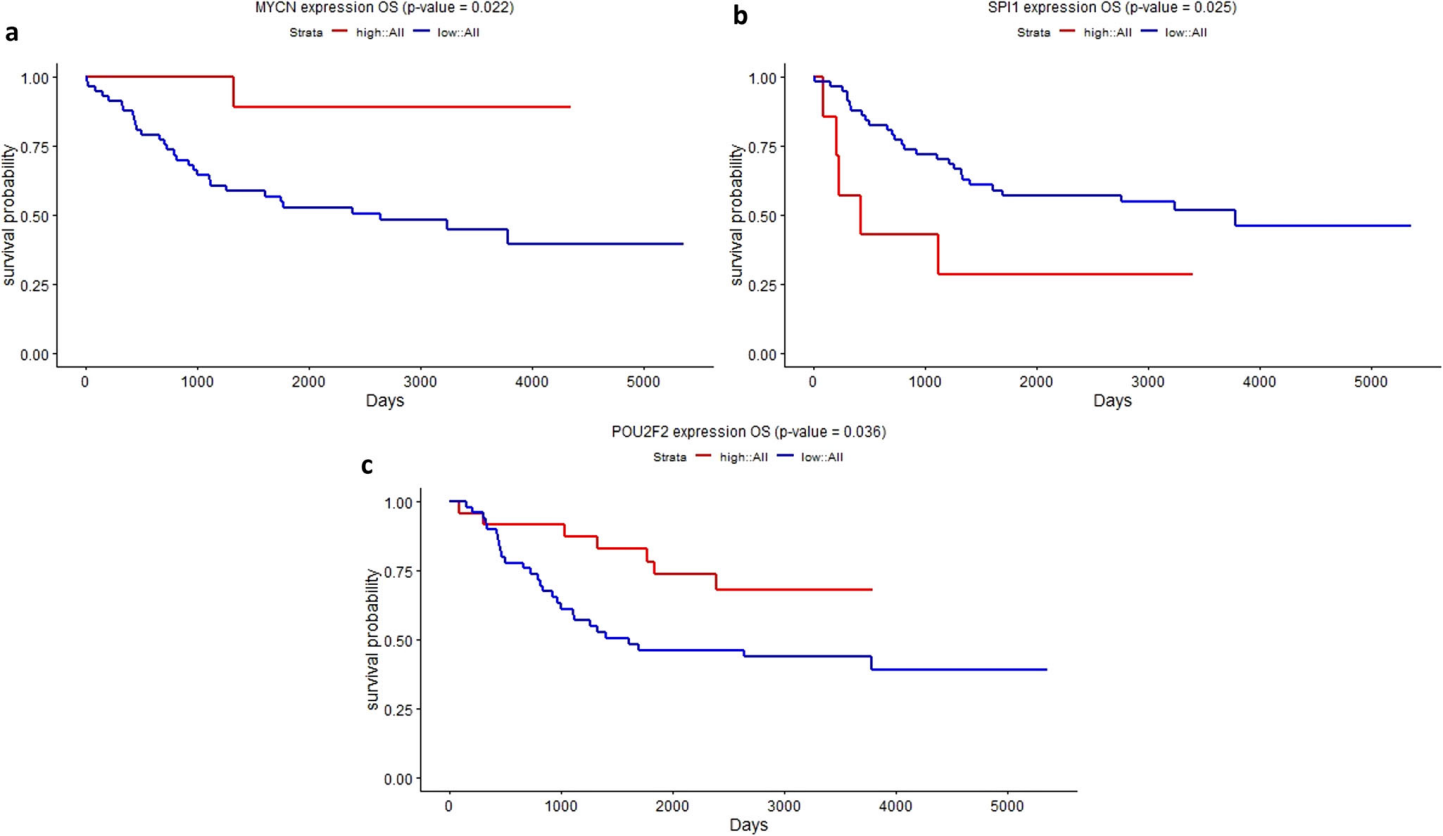
Kaplan–Meier curves for the three TFs in NB patients. **a** MYCN, **b** SPI1, and **c** POU2F2.

**Fig. 7:**
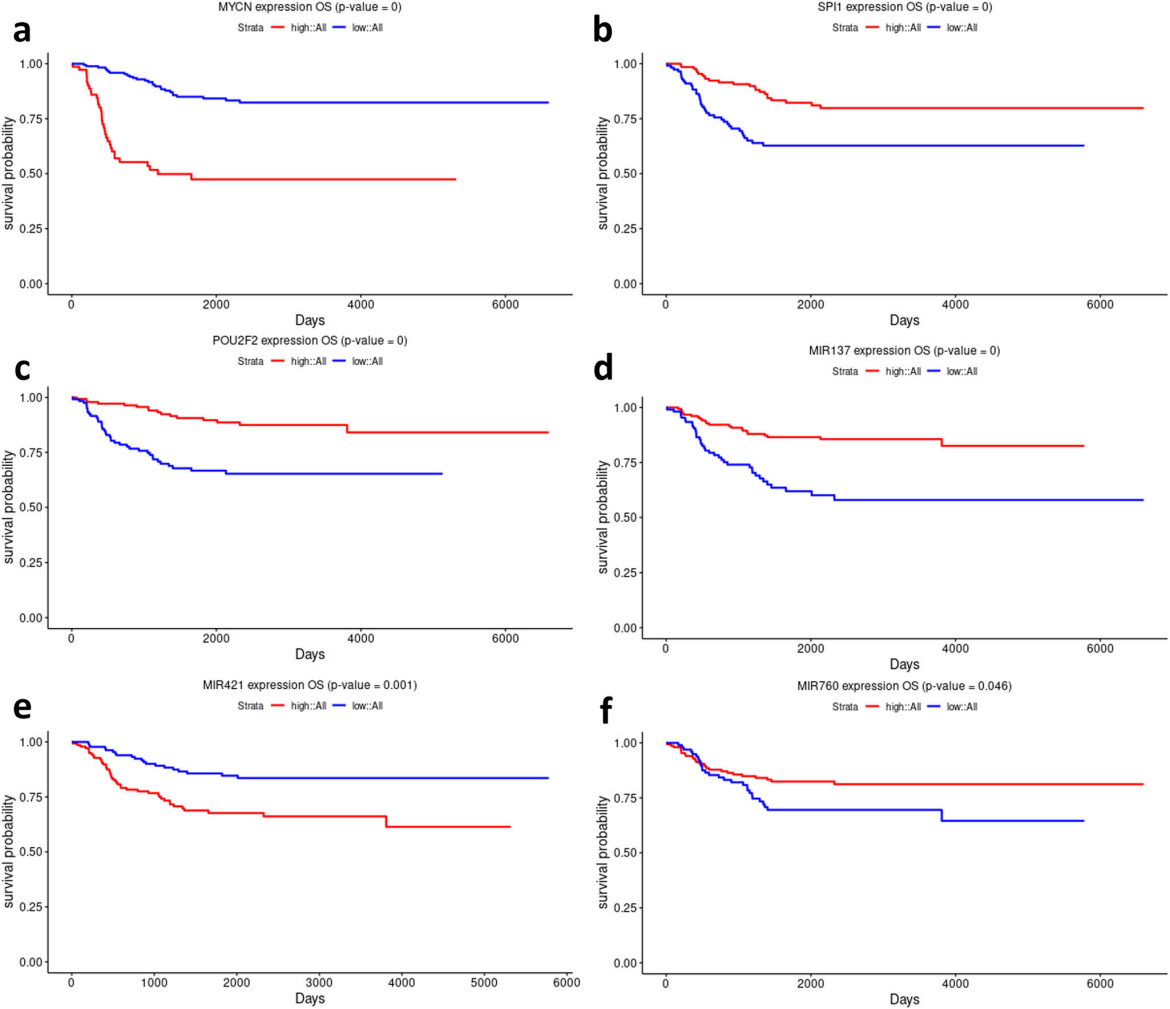
Kaplan–Meier curves of candidate biomarkers with statistical significance in the external dataset (GSE62564). **a** MYCN, **b** SPI1, and **c** POU2F2, **d** hsa-mir-137, **e** hsa-mir-421, and **f** hsa-mir-760.

Survival analysis was conducted to study the correlation between the expression of the identified hub nodes and the prognosis of NB patients. Patients were split into low- and high-expression groups based on the cutoff mean ± 0.25 * standard deviation. Nodes with *p* value < 0.05 are considered statistically significant. Table 2 illustrates the *p* values associated with the Kaplan–Meier analysis. MYCN, POU2F2, and SPI1 transcription factors demonstrated significant association with survival information, as illustrated in Fig. 6. No miRNAs have shown significant differences between the low- and high-expression groups (*p* value > 0.05). GSE62564 is an external dataset of 498 NB patients that has been utilized as a validation dataset. The three transcription factors maintained their significant association with survival information, which suggests their prognostic potential, as shown in Fig. 7a–c. Three more miRNAs hsa-mir-137, hsa-mir-421, and hsa-mir-760 showed significant association with *p* values of 7.14E-06,0.001, 0.046, respectively, as illustrated in Fig. 7d–f. Table 2 illustrates the *p* values associated with the Kaplan–Meier analysis.

**Table 2:** *P* values associated with the Kaplan–Meier curve using log-rank statistical and Chi-square tests

Nodes	MYCN	SPI1	POU2F2	hsa-mir-137	hsa-mir-421	hsa-mir-2110	hsa-mir-1305	hsa-mir-1976	hsa-mir-940	hsa-mir-760
OS *p* value (TARGET)	**0.022**	**0.025**	**0.036**	0.197	0.35	0.072	0.471	0.582	0.66	0.97
OS *p* value (GSE62564)	**1.48E-11**	**0.000495**	**1.46E-05**	**7.14E-06**	**0.001**	0.206	0.917	0.091	0.76	**0.046**
Chi-square *p* value	**0.0290**	0.999	**0.014**	**0.006**	0.316	0.894	0.271	**0.037**	**0.011**	**0.009**

Nodes with *p* value < 0.05 are highlighted in bold.

Moreover, NB patient stages were retrieved from the TARGET dataset, and a Chi-square test was conducted to evaluate the association between the identified candidate biomarker expressions and NB stages. A *p* value of <0.05 was considered statistically significant (Table 2).

**Fig. 8:**
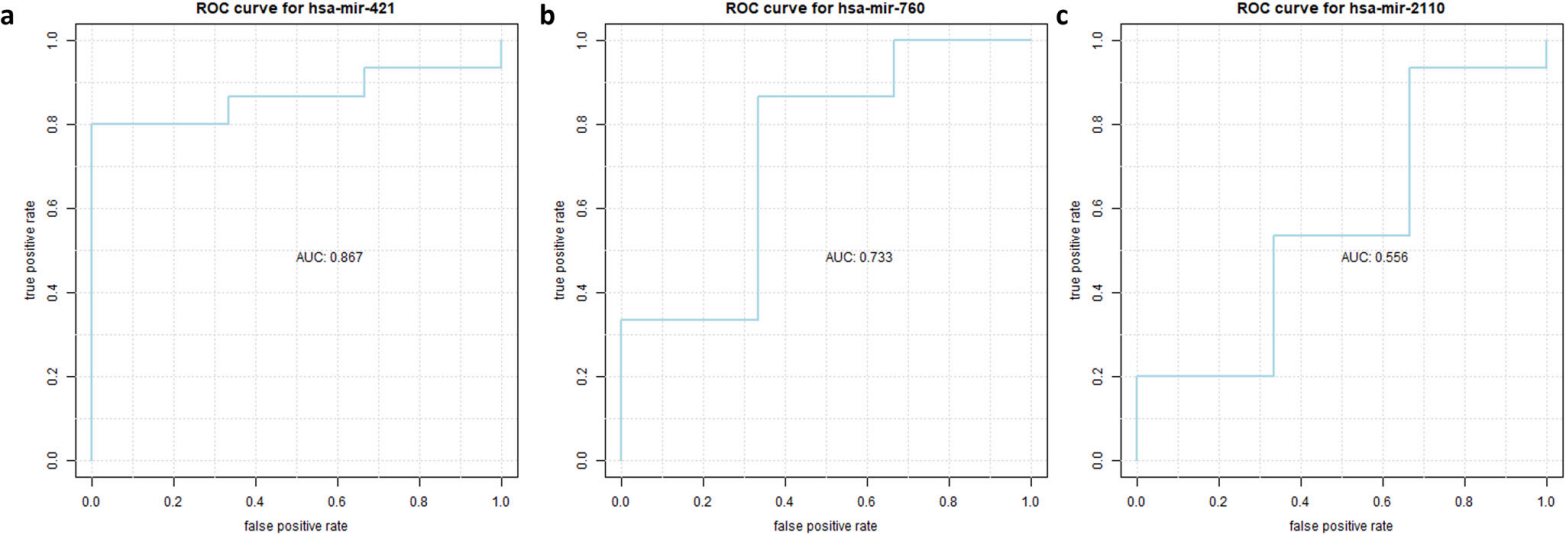
AUC values for the top three miRNAs. **a** hsa-mir-421, **b** hsa-mir-780, **c** hsa-mir-2110.

Expression profiles of miRNAs were utilized from GSE128004. The dataset consists of fifteen NB patients and three control samples. We evaluated the proposed miRNAs for their diagnostic potential. Six out of the seven miRNAs were retrieved from the dataset. hsa-mir-421 and hsa-mir-760 demonstrated a high potential to discriminate the patient group from controls with the area under the curve (AUC) values > 0.7, as shown in Fig. 8a, b. On the other hand, hsa-mir-2110 had an AUC value of 0.556 (Fig. 8c). All AUC values are listed in Table 3.

**Table 3:** AUC values for candidate miRNAs

Candidate miRNAs	hsa-mir-137	hsa-mir-421	hsa-mir-2110	hsa-mir-1305	hsa-mir-1976	hsa-mir-940	hsa-mir-760
AUC value	0.444	0.867	0.556	Not available	0.356	0.4	0.733

## Discussion

Biomarkers identification for complex diseases, including cancers, is of paramount importance in its diagnosis, prognosis, and treatment. Numerous frameworks have been proposed for NB biomarkers prediction, albeit only a few are from the perspective of utilizing multi-omics to wire transcription factors-genes-miRNAs regulatory network. Herein, we conducted an *in-silico* analysis of the NB multi-omics dataset, including mRNA, miRNA, and methylation data retrieval, integration, regulatory network construction, and analysis to identify candidate biomarkers for NB. In the constructed network shown in Fig. 5, the MCC node ranking method was utilized to determine the hub nodes (Table 1). Multiple analyses have been conducted to validate the obtained results.

Among the identified candidates, the **MYCN** gene had the highest degree of 46 and is significantly correlated with NB patient survival and NB stage. Also, its interactions with the network are shown in Supplementary Fig. 2. MYCN was found to be an amplified homolog to v-MYC but different from MYC in the human NB^29^. As illustrated, MYCN activates other hub nodes, including hsa-miR-760, hsa-miR-421, and hsa-miR-2110. For example, the upregulated hsa-miR-760 regulates another hub TF, SPI1 (Fig. 5), which in turn upregulates another identified hub mirRNA, hsa-miR-1976 (Fig. 5). These interactions between different hub nodes starting from MYCN could be implicated in NB tumor progression. Thus, its targeting could aid in inhibiting the tumorigenicity of NB. MYCN also suppresses ATM expression via its upregulation of hsa-miR-421 (Fig. 5), which promotes neuroendocrine prostate cancer cells’ invasion and migration potential^30,31^. MYCN was found to stimulate rat fibroblast cells’ S phase entry and their transformation into a more proliferative form with rapid cell-cycle progression. Similar results were observed during experimentation on growth factors-deprived quiescent fibroblasts^32–34^. As shown in Fig. 5, hsa-miR-181-a, and -b are activated by MYCN, which induces NB tumorigenesis^35^. Furthermore, it suppresses NB invasion by directly targeting non-canonical TGF-beta pathway genes^36,37^. MYCN regulates various cellular and molecular processes that support NB tumorigenicity and biomass and its expression is the highest in immature cells, which decreases the differentiation capacity of these cells. This is consistent with the NB case, as NB cell differentiation is accompanied by inhibition of MYCN expression^38–44^ and NB cells’ self-renewal and maintenance of their pluripotency properties^45–47^. Notably, NB invasion and metastasis behavior depend on the MYCN state^48,49^. Interestingly, priming of NB detachment from the extracellular matrix (ECM) for metastasis and invasion is achieved by MYCN stimulation to block integrins α1 and β1 expression^50,51^. Also, MYCN directs NB degradation of their ECM, metastasis, adhesion, and invasive behaviors via promoting focal adhesion kinase (FAK), which is responsible for integrins signaling, to stimulate migration and metastasis in tumor cells^52,53^. Intriguingly, P53 blocks the transcription of FAK, which emphasizes the role of MYCN cascades to inhibit p53^53^.

**SPI1** has an MCC score of 29 (Table 1) and is correlated with NB patient survival (Table 2). Its regulatory network shows its direct activation to hsa-miR-146a and hsa-miR-342, a repressive effect on hsa-miR-92a-1 and hsa-miR-20a, as shown in Fig. 5). SPI1 (encoding PU.1 protein) is one of the Ets transcription factors family. It is known to regulate multiple miRNAs in alignment with Supplementary Fig. 3^54^. The Ets family regulates various cellular processes, including apoptosis, proliferation, differentiation, angiogenesis, lymphoid cell development, and invasiveness^55–59^. Mice lacking PU.1 expression have neither lymphocyte, myeloid cells, nor their progenitor cells^60^. Moreover, the loss of PU.1 expression is implicated in the loss of cellular communication, leading to leukemia^61^. Also, minimal reduction in PU.1 expression primes a preleukemia state, thus promoting the development of acute myeloid leukemia^62^. It has been recently reported that PU.1 antagonizes the expression of the P53 tumor suppressor protein, thus blocking the expression of the cell cycle and apoptosis regulatory proteins activation^63^. Furthermore, the involvement of PU.1 protein in accelerating the speed of the replication elongation during the S phase of the cell cycle coinciding with an accumulation of genetic mutations and no DNA breakage in leukemia has also been reported^64^. Moreover, the PU.1 protein has been identified to promote glioma proliferation and metastasis^65^ via the regulation of Phosphoribosylaminoimidazole Carboxylase and Phosphoribosylaminoimidazolesuccinocarboxamide Synthase (PAICS) that has been recently reported to be implicated in glioma^66^, along with other malignancies development and progression^67–72^. A study reported that the expression of PU.1 protein is active in NB cells^73^. To the best of our knowledge, the involvement of PU.1 protein in NB development and progression has not been reported yet.

**POU2F2** (Oct-2) transcription factor was found to be correlated with NB patient survival and tumor stage in our analysis (Table 2) with an MCC score of 12 (Table 1). It regulates a set of miRNAs that may have a role in NB tumorigenesis (Supplementary Fig. 4). From a biological aspect, POU2F2 expression in various cancers is shown to be upregulated and consequently was proven to regulate various processes that aid cancer development. In glioblastoma, it has been reported to regulate the metabolic shift from oxidative phosphorylation to glycolysis (The Warburg effect fundamental theory^74^) via PI3K/Akt/mTOR pathway promoting tumor cells proliferation, growth, and survival^75^. It promotes lung cancer’s proliferation and mesenchymal characteristics via upregulating AGO1^76^. In NB, POU2F2 is responsible for cell development and proliferation and was reported in previous bioinformatics analyses to be hypermethylated, which drives its downregulation^77,78^. Still, more in vitro and animal model reports should be conducted to elucidate the role of the POU2F2 transcription factor in regulating NB development and progression.

Numerous reports have verified the role of microRNA networks in regulating post-transcriptional modifications of gene expression transcripts; thus, they are present in all types of tissues to regulate a broad spectrum of biological processes^79^. **Hsa-miR-137** had an MCC score of 28 (Table 1) and is correlated with the NB stage. Hsa-miR-137 represses some genes involved in NB cell fate commitment, survival, and differentiation, such as GRN, GAD2, MEGF9, SLC1A5, SLC8A1, and SCN2A as illustrated in Supplementary Fig. 5^80–84^. It is known to be enriched in the brain tissues of mice and humans, with high expression in the hippocampus and cortical brain parts and low expression in the brain stem and cerebellum^85^. Many studies reported its role in modulating cellular differentiation and proliferation in the adult brain and embryonic stages. Sun et al. reported the involvement of hsa-miR-137 in halting cellular proliferation and stimulating their differentiation^86^. The role of hsa-miR-137 in glioblastoma multiforme-derived stem cells and brain malignancies stem cells differentiation into neural lineage has also been established^87^. On the contrary, a report confirmed the hsa-miR-137 role in the induction of adult neural stem cell proliferation and halting differentiation via repressing Ezh2^88^. These reports suggest that the role of hsa-miR-137 in neural stem cell differentiation is context-dependent. Overall, these results support the role of hsa-miR-137 in regulating pluripotency to differentiation states transition and its involvement in regulating apoptosis priming; its depletion is accompanied by developmental and tumor malformations^89^. A clinical analysis of 61 NB samples unraveled that hsa-miR-137 has a substantially lower expression with other higher prognostic factors, including high mouse/murine double minute 2 (Mdm-2) and MYCN amplification^90^. It targets histone demethylase, lysine-specific demethylase 1 mRNA in NB cells, activating multiple cellular processes that coincide with tumor suppression^91^. Consequently, the re-expression of hsa-miR-137 could be a therapeutic strategy as a potential tumor suppressor.

In the case of **hsa-miR-421**, it has an MCC score of 20. It is an oncomiR that is being activated by TGFB1^92^ and MYCN as shown in Fig. 5. Hsa-miR-421 represses many neuronal-specific genes such as SOBP, HIPK, SLC43A1, FRS2, and OXR1 (Supplementary Fig. 6). Interestingly, aberrant expression of hsa-miR-421 regulates various types of malignancies via driving various genes, as shown in Fig. 5. Other than gastric cancer^93^, hsa-miR-421 overexpression has a critical role in regulating pancreatic cancer^94^, breast cancer^95^, and prostate cancer^96^. Lie et al. reported the role of hsa-miR-421 in targeting myocyte enhancer factor-2D, which inhibits glioma cell glucose-dependent processes, angiogenesis, and invasion and enhances glioma cell sensitivity to radiotherapeutics^97^. Furthermore, MYCN amplification in NB inhibits ATM cascade, a canonical cascade in DNA damage repair, apoptosis, and cell-cycle arrest, via hsa-miR-421. Hsa-miR-421 is overexpressed in NB cells compared with matched normal cells, increasing its proliferation potential, migration, cell-cycle progression, and invasion capacity via targeting the tumor suppressor, Menin^98^. This is due to the inducing role of the TGFB1 transcription factor^99^.

**Hsa-mir-2110** has previously been reported to act as a tumor suppressor. Zhao et al. reported the role of eight of thirteen differentiation-stimulating miRNAs, including hsa-miR-2110, in direct or indirect blocking MYCN expression. Furthermore, it was observed that the expression levels of hsa-mir-137 and hsa-miR-2110 exhibit a substantial inverse correlation with the levels of MYCN, emphasizing their interaction with MYCN mRNA in NB progression^100^. Its effect on NB cell line survival has been reported with varied differentiation induction methodologies. Furthermore, they identified the clinical relevance of Tsukushi expression and hsa-miR-2110, where the higher Tsukushi mRNA level is associated with a low survival rate. Another report shows the oncosuppressive role of hsa-miR-2110 in NB via targeting Tsukushi mRNA and inhibiting NB tumorigenesis^101^. Zhang et al. investigated the inhibitory effect of upregulating hsa-miR-2110 on triple-negative breast cancer cell proliferation, invasion, and migration in vitro and tumor growth in vivo^102^. As shown in Supplementary Fig. 7, MYCN prime hsa-miR-2110 expression, which represses the inhibitor of differentiation −3 (ID3), which has a critical role in sustaining cellular proliferation and halting the differentiation process^103,104^.

**Hsa-miR-1305** has an MCC score of 18 (Table 1) and is known as a tumor suppressor via regulating Mdm-2. As shown in Supplementary Fig. 8, hsa-miR-1305 has a repressive effect on CREBBP. It also represses the THADA fusion gene, which is proven to trigger insulin-like growth factor 2 (IGF-2) mRNA binding protein 3, which is known to be overexpressed in cancer^105^. Our network showed that hsa-miR-1305 downregulates the COL12A1 gene, which encodes for collagen type XII α1 that is found to trigger tumor progression and metastasis^106–108^. Hsa-miR-1305 also represses TUBB, which encodes for the beta-tubulin protein that is broadly expressed in the neuronal cells, which ultimately induces NB tumorigenesis (Fig. 5)^109^. Cai et al. investigated the effect of suppressing hsa-miR-1305 expression on the aggressiveness of non-small cell lung cancer cells (NSCLC), showing upregulation hsa-miR-1305 target Mdm-2, a p53 antagonist, which in role increases p53, a tumor suppressor, inducing attenuation of NSCLC^110^. Hsa-miR-1305 has been reported to have a critical role in circCOG2-mediated colorectal cancer proliferation, migration, and invasion via TGFB2/Smad pathway^111^. The role of exosomal hsa-miR-1305 has been established in inducing multiple myeloma tumorigenesis via decreasing cellular hsa-mir-1305, thus increasing the abundance of its target genes such as IGF1, FGF2, and Mdm-2^112^. Another report investigated the effect of circRIP2 on targeting hsa-miR-1305 to activate the TGF-β2/Smad3 cascade to induce epithelial to mesenchymal transition (EMT), accelerating bladder cancer aggressiveness^113^. It has been reported that hsa-miR-1305 silencing significantly inhibits hepatitis B virus-associated hepatocellular carcinoma tumorigenesis^114^. Despite numerous previous reports, hsa-miR-1305 role in NB tumorigenesis has not been studied. The diagnostic potential of hsa-miR-1305 as a biomarker could not be validated due to the limited availability of NB datasets that included expression data for this specific miRNA.

**Hsa-miR-1976** is correlated with the NB stage, as shown in Table 2. It is shown to repress the LMTK3 transcription factor while regulating SPI1 and CREBBP (Supplementary Fig. 9). Hsa-miR-1976 is downregulated in tumor tissues, where its lower expression correlates with worse overall survival in a patient cohort obtained from the TCGA database. Hsa-miR-1976 knockdown in triple-negative breast cancer markedly promotes EMT and cancer stemness via direct regulation of phosphatidylinositol-4,5-bisphosphate 3-kinase catalytic subunit gamma^115^. Chen et al.^116^ reported the downregulation of hsa-miR-1976 in NSCLC, and its upregulation repress tumorigenesis via targeting phospholipase C epsilon 1. Another report tackles the association between the abundance of hsa-miR-1976 in plasma samples and the clinicopathological features of breast cancer patients and its role as a non-invasive biomarker for breast cancer diagnosis^117^. Thus, hsa-miR-1976 could be implicated in NB tumorigenesis and be a potential biomarker.

**Hsa-miR-940** directly represses some genes associated with the neuronal differentiation of NB cells, such as TUBB, SLC1A5, MEGF9, PIP4K2A, and MYO7B (Supplementary Fig. 10). Hsa-miR-940 regulates cell survival and cancer resistance to chemotherapeutics by targeting the MAPK1 cascade^118^. Zhang et al. reported its abundance effect in promoting breast cancer via regulating FOXO3^119^. On the other hand, hsa-miR-940 is dramatically silenced in glioma cells, and its upregulation functions as a glioma suppressor via targeting cyclin kinase subunit 1, showing its tumor suppression effect^120^. Another study reported its inhibitory effect on glioma tumorigenesis via targeting methylenetetrahydrofolate dehydrogenase, which blocks folate metabolism in mitochondria^121^. Xu R. et al. reported hsa-mir-940 implication in halting EMT of glioma cells via targeting ZEB2^122^. Other than being a salivary biomarker for pancreatic cancer^123^, it also marks breast cancer, prostate cancer^124^, and esophageal squamous cell carcinoma^125^. While the involvement of hsa-miR-940 in NB development remains unreported, our analysis revealed a significant correlation between its expression and NB stage.

**Hsa-miR-760** is a hub node in our analysis with an MCC score of 11 (Table 1). As shown in Supplementary Fig. 11, it is upregulated by the MYCN gene and regulates the SPI1 transcription factor. In addition, it represses the PLD6 gene (phospholipase D), which facilitates many cellular processes in cancer progression, metabolism, and growth. It has been reported that the PLD6 gene alters mitochondrial fusion and fission dynamics, which dysregulate cellular mitochondrial bioenergetics, consequently, the progression of breast cancer^126^. Furthermore, hsa-miR-760 is downregulated in various cancer types, such as breast, prostate, NSCLC, gastric, and hepatocellular carcinoma, while upregulated in ovarian cancers^127^. Moreover, it is proven that hsa-miR-760 inhibits chemotherapeutics resistance in hepatocellular carcinoma via Notch1/Akt pathway^128^. In bladder cancer, it was found that METTL1 indirectly degrades tumor suppressor ATF3 mRNA mediated by miR-760, promoting tumor progression^129^. In our analyses, hsa-miR-760 shows promising prognostic and diagnostic ability. It is also correlated with the NB stage (Table 2, Fig. 7, and Fig. 8). However, we did not find any reports about its association with NB.

In conclusion, the proposed computational framework aims at integrating multi-omics data to identify potential biomarkers in NB. After integrating mRNA-seq, miRNA-seq, and methylation array data using SNF, feature selection was utilized to determine high-rank features. Interactions between high-rank genes and miRNAs were retrieved to build a regulatory network. It consisted of TF-miRNA interactions as well as miRNA-target interactions. We analyzed the interaction network using MCC and identified ten candidate NB biomarkers, three transcription factors, and seven miRNAs. Among them, the roles of MYCN, hsa-miR-137, hsa-miR-421, and hsa-miR-2110 in NB tumor development and progression have been studied and proven. On the other hand, the rest of the predicted biomarkers in our study, such as SPI1, POU2F2, hsa-miR-1305, hsa-miR-1976, hsa-miR-940 and hsa-miR-760, could serve as potential biomarkers to halt NB tumorigenicity. In addition, their role in other tumor development and progression has been studied. Our regulatory network shows that they interact with some well-studied NB biomarkers, including MYCN, which support their under-studied implication in NB development. Furthermore, their potential use as diagnostic biomarkers and correlation with NB patients’ survival and stage information were studied. Six out of our 10 candidate biomarkers were shown to be correlated with the INSS stage, namely MYCN, POU2F2, hsa-mir-137, hsa-mir-1976, hsa-mir-940, and hsa-mir-760. High vs. low expression of MYCN, SPI1, and POU2F2 were shown to be correlated with significantly different survival outcomes, both in our dataset and in the external validation dataset. Furthermore, survival analysis of the external validation dataset suggested hsa-mir-137, hsa-mir-421, and hsa-mir-760 could be potential prognostic markers. The receiver operating characteristic (ROC) analysis on another external validation dataset suggested that hsa-mir-421 and hsa-mir-760 may also be diagnostic markers. Thus, experimental validation of the roles of SPI1 and POU2F2 in NB tumor progression could aid biomarker discovery. Furthermore, priming the transcriptional expression of miR-1976 and targeting hsa-miR-1305, hsa-miR-940, and hsa-miR-760 could block NB tumorigenesis. Therefore, this study proposes a deeper understanding of MYCN interactome, providing candidate routes for targeted therapies in NB.

## Methods

### Data retrieval

Multi-omics data were obtained for solid tumor samples from 99 patients. The patients’ overall survival information and cancer staging according to the INSS were retrieved as well. Three data types were downloaded from the Therapeutically Applicable Research to Generate Effective Treatments (TARGET) program website. First, methylation array data were obtained using the Illumina Infinium Human Methylation 450 Platform. Methylation array data included beta values for 485577 features (reporter IDs) and mean detection *p* values. mRNA-seq data were obtained using the Illumina HiSeq2000 sequencing system. The dataset comprised FPKM values for 56038 features. Finally, the miRNA-seq data was generated using the Illumina-NextSeq500 system that included 1870 features (miRNAs). Each data type was arranged into a feature-by-patient table. Links to the methylation array, mRNA-seq, and miRNA-seq data are available in the project GitHub repository (https://github.com/ComputationalBiologyLab/Biomarker-discovery-in-Neuroblastoma-Multiomics).

### Data preprocessing

The quality of the methylation array data was assessed by confirming that all samples had a mean detection *p* value < 0.005. Around 20% of methylation array beta values had Na values. That was due to a common SNP being found within 10 bp from the CpG site or, overlapping with a repeat element within 15 bp from the CpG site, or the detection *p* value of the sample being above 0.05. These rows were removed. Reporter IDs that mapped to multiple sites were also filtered. Features that mapped to the X or Y chromosomes were removed from methylation array data and mRNA data to avoid selecting biomarkers related to sex^130^. Features that had zeros across all samples were deleted from all datasets. mRNA FPKM values were transformed to TPM values to accommodate comparison across samples^131^. The total number of features before and after preprocessing is shown in Table 4. Min-max normalization was then applied to all datasets.

**Table 4:** The number of features before and after the preprocessing stage

Data type	Methylation array	mRNA-seq	miRNA-seq
Number of original features	485,577	56,038	1870
Number of features after preprocessing	379,522	50,501	1594

### Data integration

SNF is a statistical network model that integrates the different omics data. A similarity network is created for each data type, in which a vertex represents each sample (i.e., patient), and the degree of similarity between samples is represented by weighted edges that connect the vertices. Therefore, a complete patient similarity matrix is constructed using edge weights. A sparse kernel matrix is also constructed where edge weights are used for determining the K-nearest neighbors. This operation sets the pairwise similarities of non-neighboring points to zero. Utilizing both matrices, network fusion is performed by updating the similarity matrix iteratively. After a specified number of iterations sufficient for convergence, the resultant similarity matrices are averaged to obtain the fused matrix.

SNF has the advantage of extracting valuable information even from small numbers of samples. Integrating similarity networks derived from different data types preserves both common and complementing aspects of these similarity networks, therefore showing the contribution of each data type to the overall similarity network. SNF has been used to study five types of cancer, namely glioblastoma multiforme, breast invasive carcinoma, lung squamous cell carcinoma, colon adenocarcinoma, and kidney renal clear cell carcinoma. For each cancer type, SNF was used to integrate methylation, mRNA, and miRNA expression data. On data integration, the researchers identified different subtypes that were shown to have distinct clinical hallmarks^132^.

SNF uses three hyperparameters: *T* is the number of fusion iterations, *k* is the number of nearest neighbors to consider when building the sparse kernel matrix, and *α* is a hyperparameter used in the scaled exponential similarity kernel, which is used to calculate pairwise patient similarity values. SNF convergence was independently assessed for each hyperparameter by iteratively changing the value and calculating the relative change between fused graphs obtained in consecutive iterations using the spectral norm. Both *T* and *k* were selected from the range 2:50, while *α* was chosen from the range 0.05:1 with a step of 0.05. Based on the selected values (*T* = 15, *k* = 20, and *α* = 0.5), The package “SNFtool” (https://CRAN.R-project.org/package=SNFtool) was used to perform the SNF model on the three data types, producing a fused similarity matrix.

### Feature selection

The SNF feature selection technique was utilized to assign ranks to features based on how informative they were to the developed fused network. rSNF was performed by building a patient similarity matrix based solely on each feature. Spectral clustering^133^ was performed on the single-feature-based and fused-based similarity matrices to identify disease subtypes. Then, the normalized mutual information (NMI) score^134^ was utilized to determine the concordance between the results of both clustering. The higher the score, the more important the feature contribution to the fused similarity network. Different numbers of clusters have been tried (2:7).

The ratio between the minimum intra-cluster similarity and the maximum inter-cluster similarity was employed to evaluate the clustering quality. A good clustering should follow the principles of homogeneity and separation^135^. They state that elements within the same cluster should be close to each other (homogeneity), while elements in different clusters should be distant (separation). This means that patients within the same cluster would be highly similar, whereas patients in different clusters would have a low similarity.

If the minimum similarity within a cluster is high, it ensures that all other similarities within each cluster are even higher, which helps to group similar patients together. This is achieved by calculating pairwise similarities between all samples in each cluster separately and selecting the minimum similarity across all clusters afterward. Moreover, a low maximum similarity between patients in different clusters ensures that patients within different clusters are even less similar, making the clustering more reasonable. This is done by calculating pairwise similarities between all samples in different clusters in which we pick the maximum value.

Therefore, a similarity measure of the ratio between the minimum intra-cluster similarity (within) and the maximum inter-cluster similarity (between clusters) has been utilized to compare the different numbers of clusters to identify which is the best in terms of compactness and well separation. We have selected the number of clusters that achieved the highest ratio (*c* = 4).

Our approach is similar to the Dunn index^136^, which measures the quality of clustering. However, the Dunn index utilizes distances between samples, while we use sample similarities. The similarity measure we used is calculated as follows:

1 $$\nabla \left({c}_{k}\right)={\min }_{{x}_{i},{x}_{j}\in {c}_{k}}S\left({x}_{i},{x}_{j}\right)\forall {x}_{i},{x}_{j}\in {c}_{k},i\ne j,\forall k=0,1,2,\ldots .,n$$

Where dataset *D* with $m$ samples, include ${x}_{1},{x}_{2},{x}_{3},\ldots .{x}_{m}$, $n$ is the number of clusters in the cluster set C, $\nabla ({c}_{k})$ denotes intra-cluster similarity of a cluster, and $S$ is the similarity between two samples. That will result in k values where each one represents the inter-cluster similarity for each cluster as shown in Eq. 1. The inter-cluster similarity between clusters $\triangle \left({c}_{k},{c}_{r}\right)$ is calculated as illustrated in Eq. 2.

2 $$\triangle \left({c}_{k},{c}_{r}\right)={\max }_{{x}_{i}\in {c}_{k},{x}_{j}\in {c}_{r}}S\left({x}_{i},{x}_{j}\right)\forall {x}_{i}\in {c}_{k},{x}_{j}\in {c}_{r},i\ne j,k\ne r,\forall k=\mathrm{0,1,2},\ldots .,n$$

The clustering quality ratio $Q$ is calculated as follows:

3 $$Q\left(C\right)=\frac{{\min }_{{c}_{k}\in C}\nabla ({c}_{k})}{\triangle \left({c}_{k},{c}_{r}\right)}\forall k=\mathrm{0,1,2},\ldots .,n$$

The features were ranked based on their NMI score. For all three data types, it has been noticed that, after the initial few features, there was a consistent decline in the NMI score. The remaining features had negligible changes (Supplementary Fig. 12). To prioritize the most informative features for the fused network, the top 10% were chosen (Supplementary Data 1–3). Both mRNA-seq and methylation array features were then mapped to gene symbols, and shared genes were selected as essential genes along with the high-rank miRNAs for subsequent analysis. The complete lists of high-rank genes are illustrated in Supplementary Data 4.

### Regulatory network construction and analysis

To gain a better understanding of the identified essential genes and high-rank miRNAs, possible interactions between the two sets were explored. To build an interaction network, interaction data for these biological molecules were obtained. Two types of gene-miRNA interaction data were retrieved. TF-miRNA regulations were acquired from the transmir 0.2 database^137^, and miRNA-target interactions were acquired from the Tarbase v8 database. The transmir 0.2 database is a manually curated database with comprehensive information about the TF-miRNA regulations. The genes encoding for transcription factors in the candidate essential genes were identified through the database. Transmir 0.2 database includes experimentally verified interactions with different levels of confidence. Level 2 interactions were predicted by ChIP-seq experiments and further verified through high-throughput experiments, while literature-based interactions were manually curated from scientific papers. Level 2 and literature-based interactions were retrieved in our study. On the other hand, the Tarbase v8 database contains experimentally supported miRNA-target interactions. From both databases, interactions found in humans were downloaded. Interactions involving the identified high-rank features were extracted for further analysis.

Further annotations were acquired from the transmir 0.2 database website and added to our interaction tables. Transcription factors and miRNAs were annotated with associated diseases. Transcription factors were further annotated with prognostic correlations. The complete interaction and attribute tables are illustrated in Supplementary Data 5 and 6.

The regulatory network between TFs, genes, and miRNAs was constructed using Cytoscape software (version 3.9.1) after filtering normal cell line interactions. Node shape, color, edge shape, and type (activation or repression) were modified for visualization. Network analysis was employed to evaluate and rank the hub TFs, genes, and miRNAs. MCC algorithm in the Cytoscape plugin, CytoHubba, was utilized to identify the hub nodes in the regulatory network as it showed high-quality performance and accuracy over other measures, including radiality, stress, clustering coefficient, and betweenness^138–142^.

### Analysis of the identified candidate biomarkers’ association with NB

Survival analysis was performed using the Kaplan–Meier method with a log-rank statistical test to explore the association of the proposed biomarkers with the overall survival of NB patients and identify which will be of prognostic potential. Biomarkers with *p* value < 0.05 are considered to have prognostic potential. Another external dataset, GSE62564, has been utilized to validate the results. The survival R package^143^ was used for this purpose. The correlation between potential biomarker expressions and cancer stages was also statistically assessed using the Chi-square test. A *p* value < 0.05 was considered significant.

The ROC curve analysis was utilized to examine the diagnostic potential of the identified miRNAs by assessing their ability to discriminate between NB patients and normal samples. pROC R package^144^ was used to generate ROC curves and calculate the AUC values for the proposed biomarkers from the GEO dataset GSE128004.

### Reporting summary

Further information on research design is available in the Nature Research Reporting Summary linked to this article.

### Supplementary information

Supplementary Figures

Reporting summary

Data 2

Data 1

Data 3

Data 4

Data 5

Data 6

## Data Availability

Publicly available datasets were analyzed in this study. All the data used in this study are derived from the TARGET database (https://target-data.nci.nih.gov/) under TARGET Neuroblastoma (NBL) project. Datasets used in validation were retrieved from Gene Expression Omnibus (GEO) database under accession numbers GSE62564 and GSE128004 and are available at the following URLs: https://www.ncbi.nlm.nih.gov/geo/query/acc.cgi?acc=GSE62564, https://www.ncbi.nlm.nih.gov/geo/query/acc.cgi?acc=GSE128004.

## Code availability

The code for reproducing the transcriptomic analysis results is available in the GitHub repository. See Code https://github.com/ComputationalBiologyLab/Biomarker-discovery-in-Neuroblastoma-Multiomics.
