# Supplementary material for: A multi-omics approach for biomarker discovery in neuroblastoma: a network-based framework
Source: NPJ Syst Biol Appl. 2024 May 17;10:52. doi: 10.1038/s41540-024-00371-3 (PMC11101461; doi:10.1038/s41540-024-00371-3)
Supplement: Supplementary file 1 — Supplementary Figures [file 41540_2024_371_MOESM1_ESM.pdf]

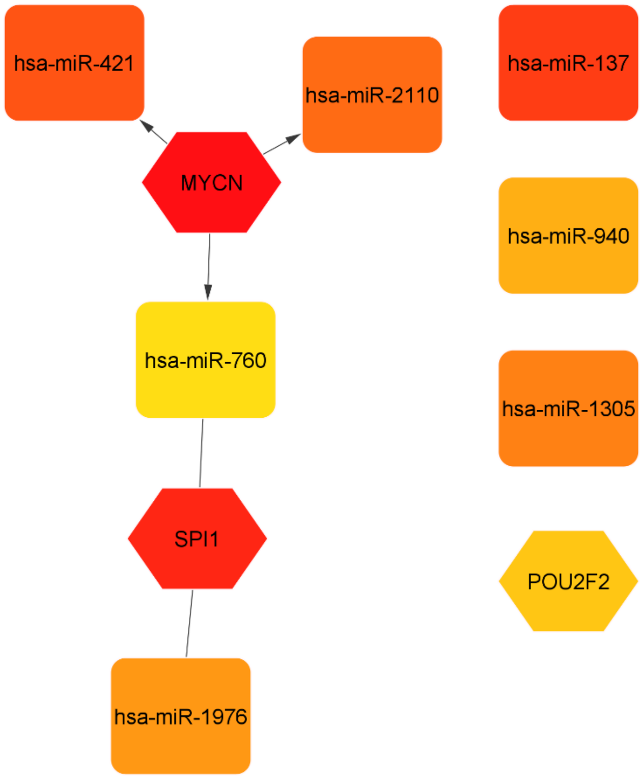

Supplementary Fig. 1 An interaction network between the top ten hub nodes. The color is degraded from red to yellow according to their MCC ranking score.

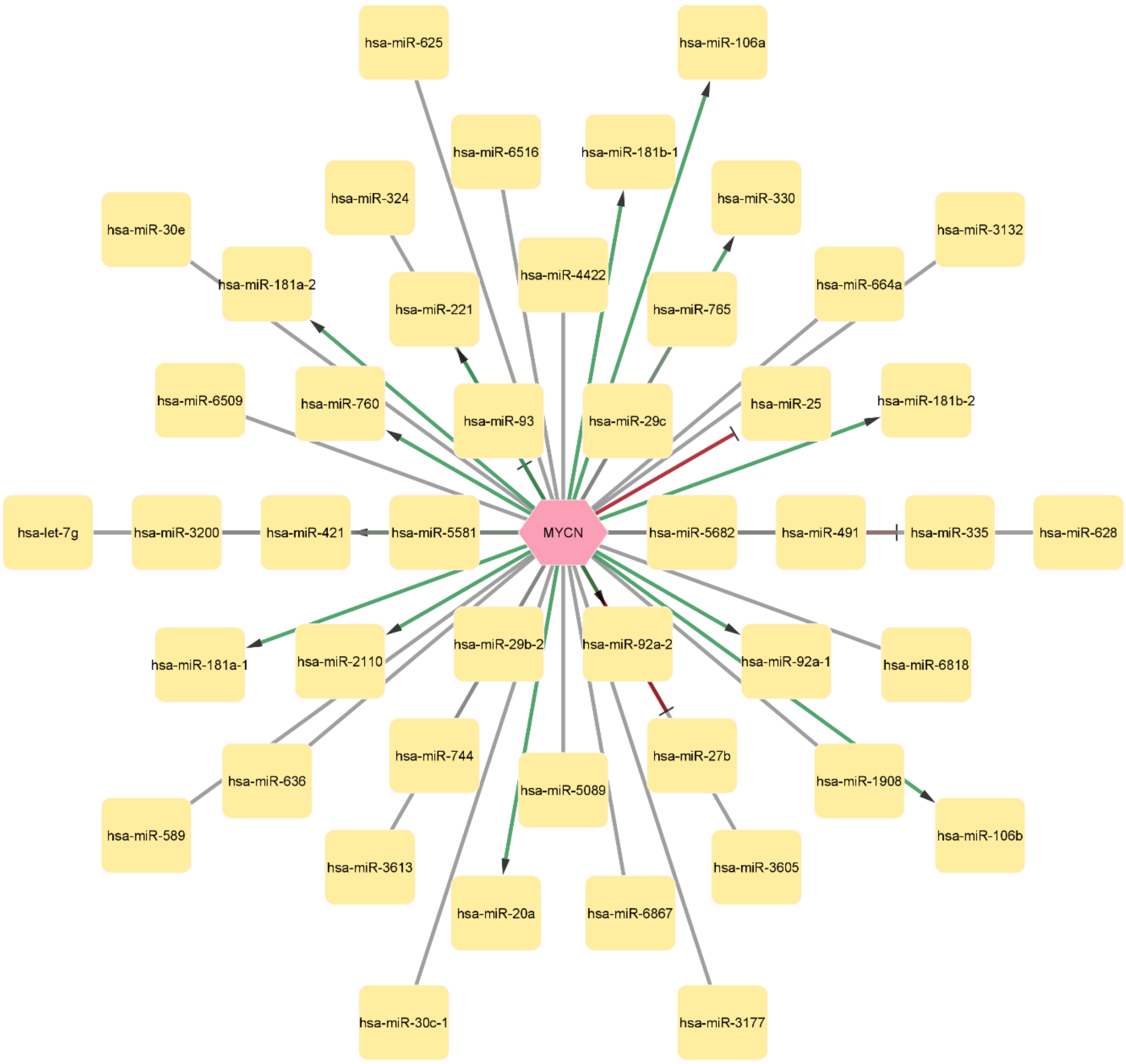

Supplementary Fig. 2 MYCN transcription factor network interactions with various miRNAs show that the MYCN regulatory network consists of 47 nodes and 46 edges. Node shape and color are labeled as pink hexagon indicates TFs and yellow round square indicates miRNAs. The nodes have colored and directed edges: T-shaped red edges indicate repression, delta-shaped green edges indicate activation, and line edges indicate regulation. Black colored edges indicate regulation.

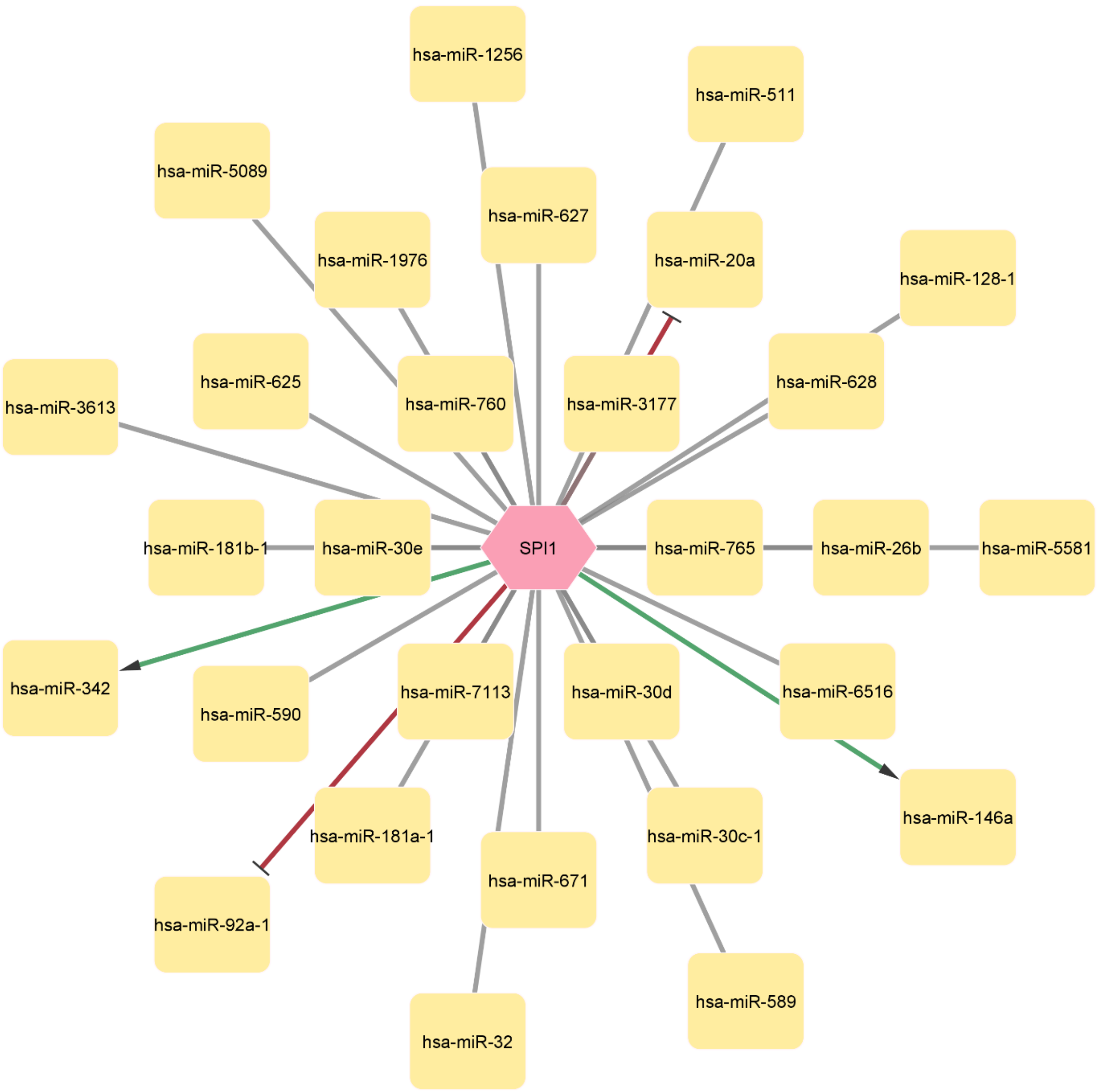

Supplementary Fig. 3 SPI1 transcription factor network interactions with various miRNAs show that the SPI1 regulatory network consists of 30 nodes and 29 edges. Node shape and color are labeled as pink hexagon indicates TFs and yellow round square indicates miRNAs. The nodes have colored and directed edges: T-shaped red edges indicate repression, delta-shaped green edges indicate activation, and line edges indicate regulation. Black colored edges indicate regulation.

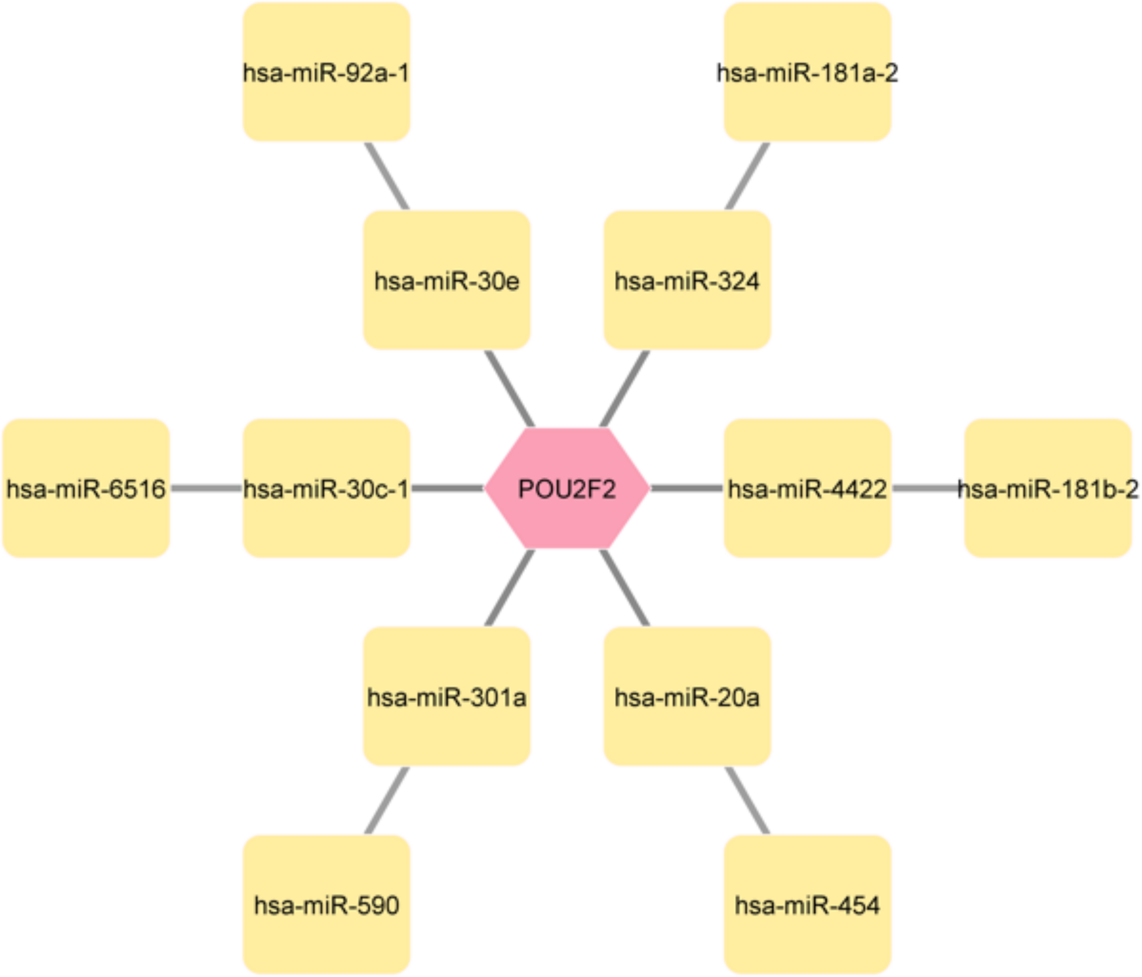

Supplementary Fig. 4 POU2F2 transcription factor network interaction with various miRNAs consists of 13 nodes and 12 edges. Node shape and color are labeled as pink hexagon indicates TFs and yellow round square indicates miRNAs. The nodes have colored and directed edges: T-shaped red edges indicate repression, delta-shaped green edges indicate activation, and line edges indicate regulation. Black colored edges indicate regulation.

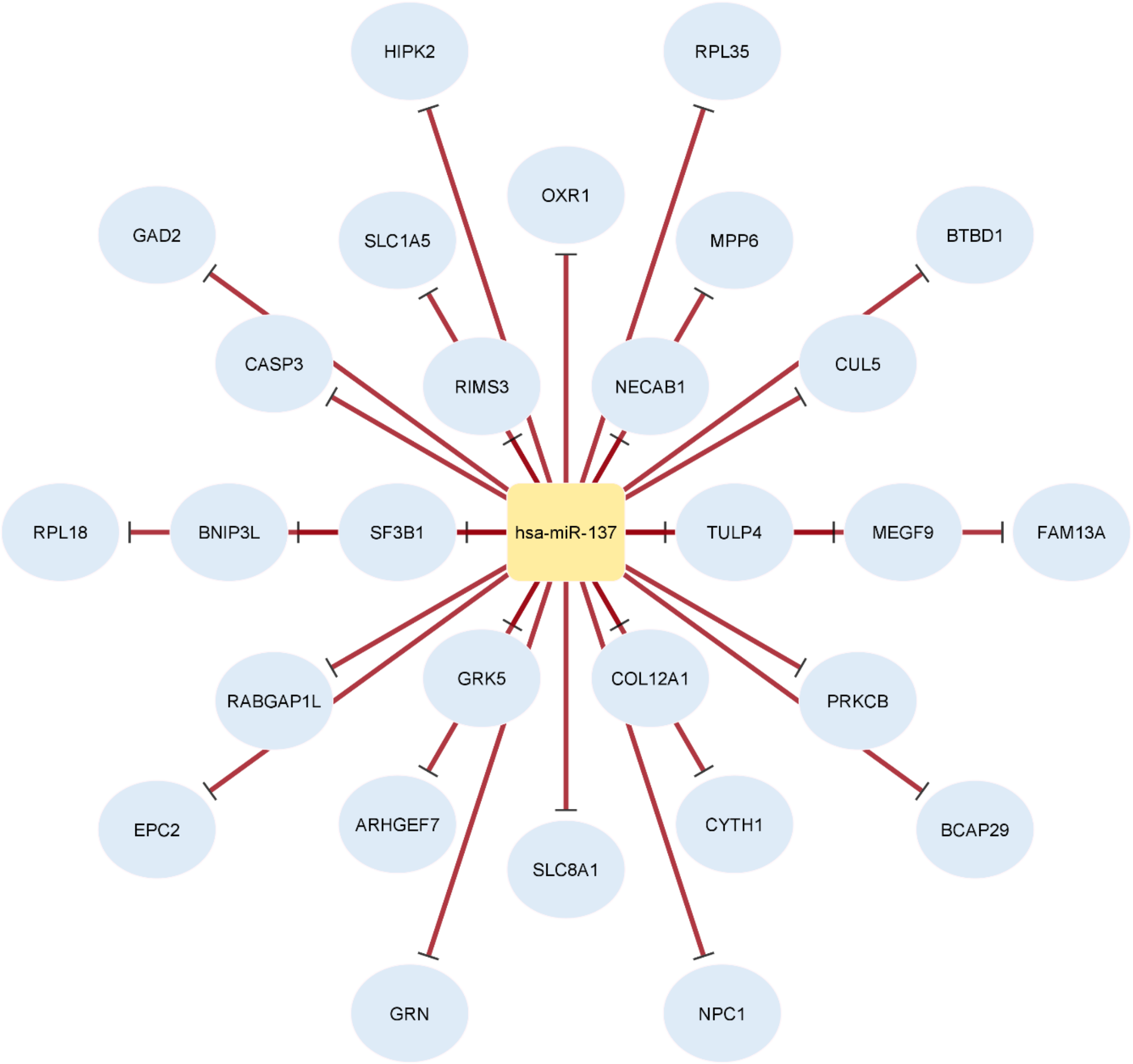

Supplementary Fig. 5 Regulatory networks of hsa-miR-137. Node shape and color are labeled as pink hexagons representing TFs, blue ellipses representing genes, and yellow round squares representing miRNAs. The nodes have directed and colored edges: the delta-shaped green arrows indicate upregulation, while the T-shaped red arrows indicate downregulation.

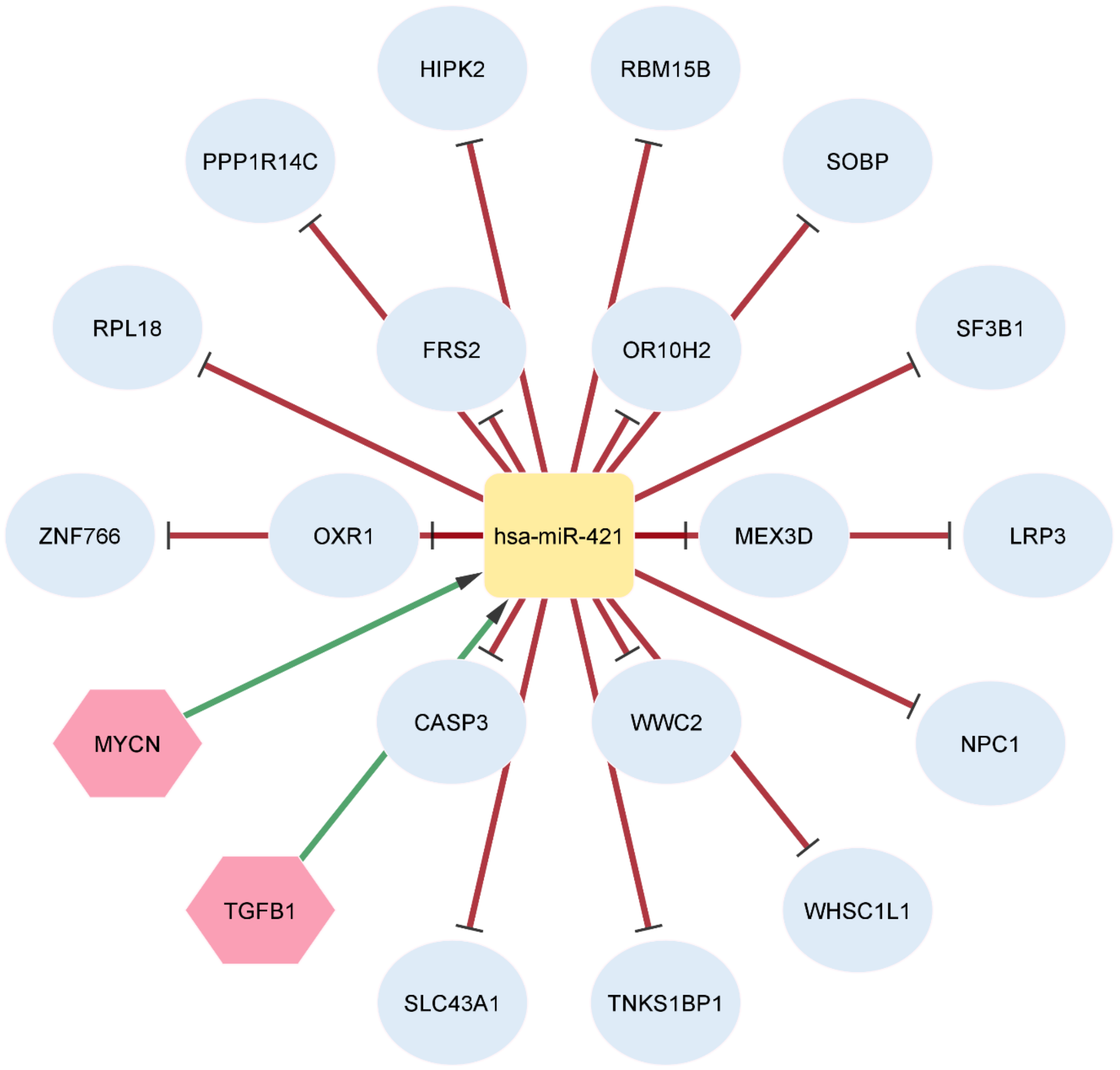

Supplementary Fig. 6 Illustration of hsa-miR-421 interactions. Node shape and color are labeled as pink hexagons representing TFs, blue ellipses representing genes, and yellow round squares representing miRNAs. The nodes have directed and colored edges: the delta-shaped green arrows indicate upregulation, while the T-shaped red arrows indicate downregulation.

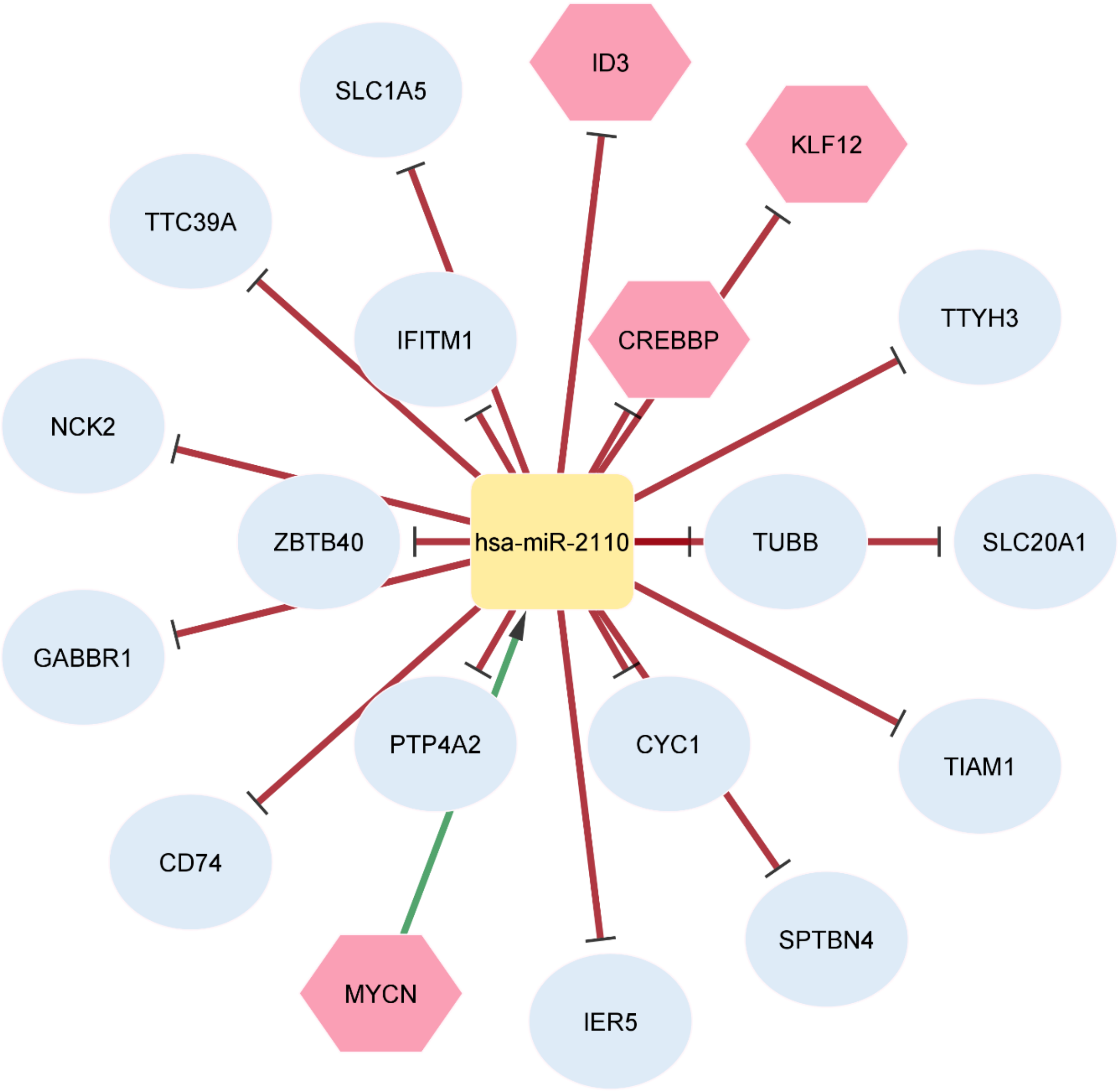

Supplementary Fig. 7 hsa-miR-2110 interactions with other nodes of the regulatory network. Node shape and color are labeled as pink hexagons representing TFs, blue ellipses representing genes, and yellow round squares representing miRNAs. The nodes have directed and colored edges: the delta-shaped green arrows indicate upregulation, while the T-shaped red arrows indicate downregulation.

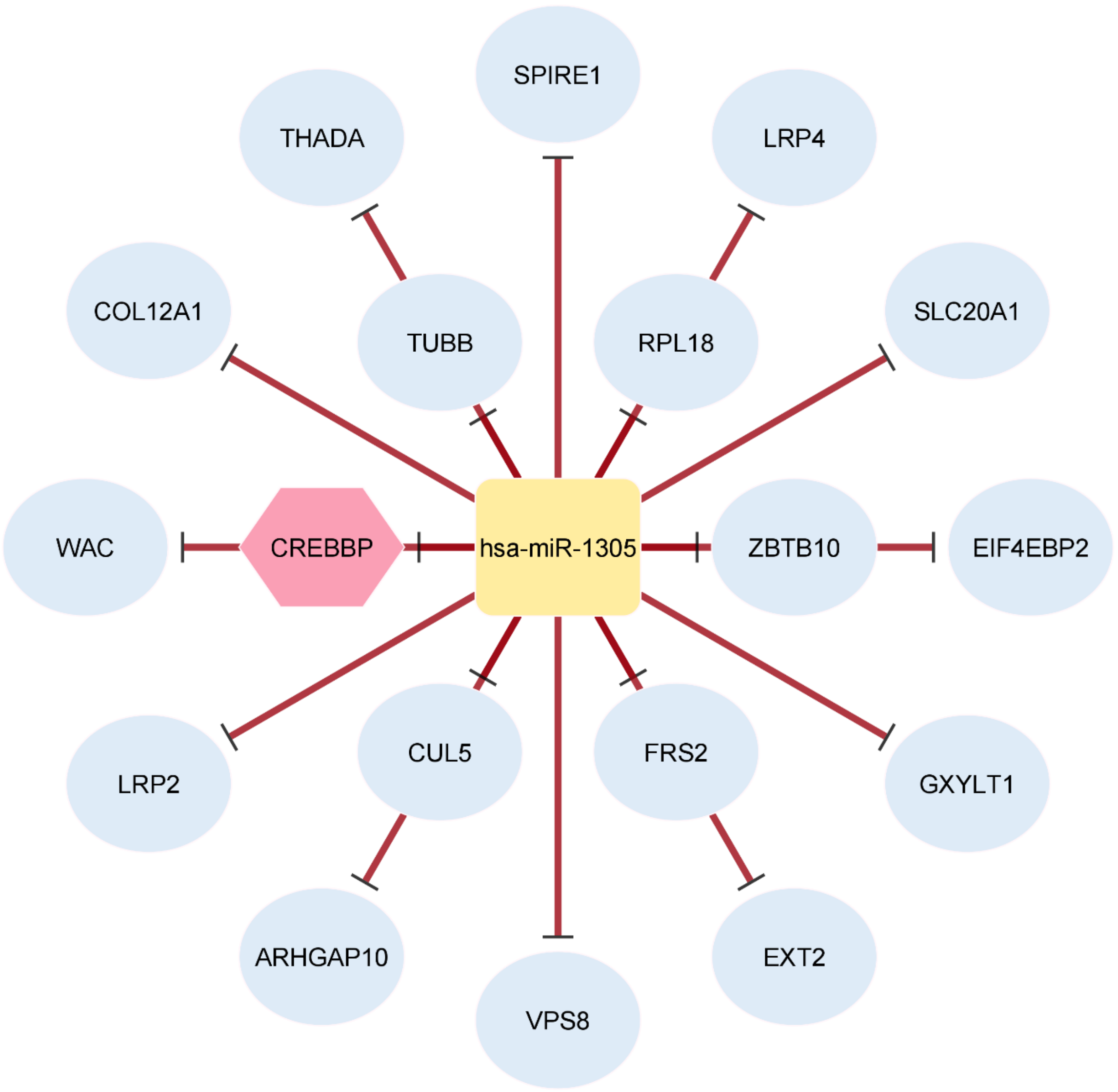

Supplementary Fig. 8 The regulatory network of hsa-miR-1305 consisting of 19 nodes and 18 edges. Node shape and color are labeled as pink hexagon indicates TFs and yellow round square indicates miRNAs. The nodes have colored and directed edges: T-shaped red edges indicate repression.

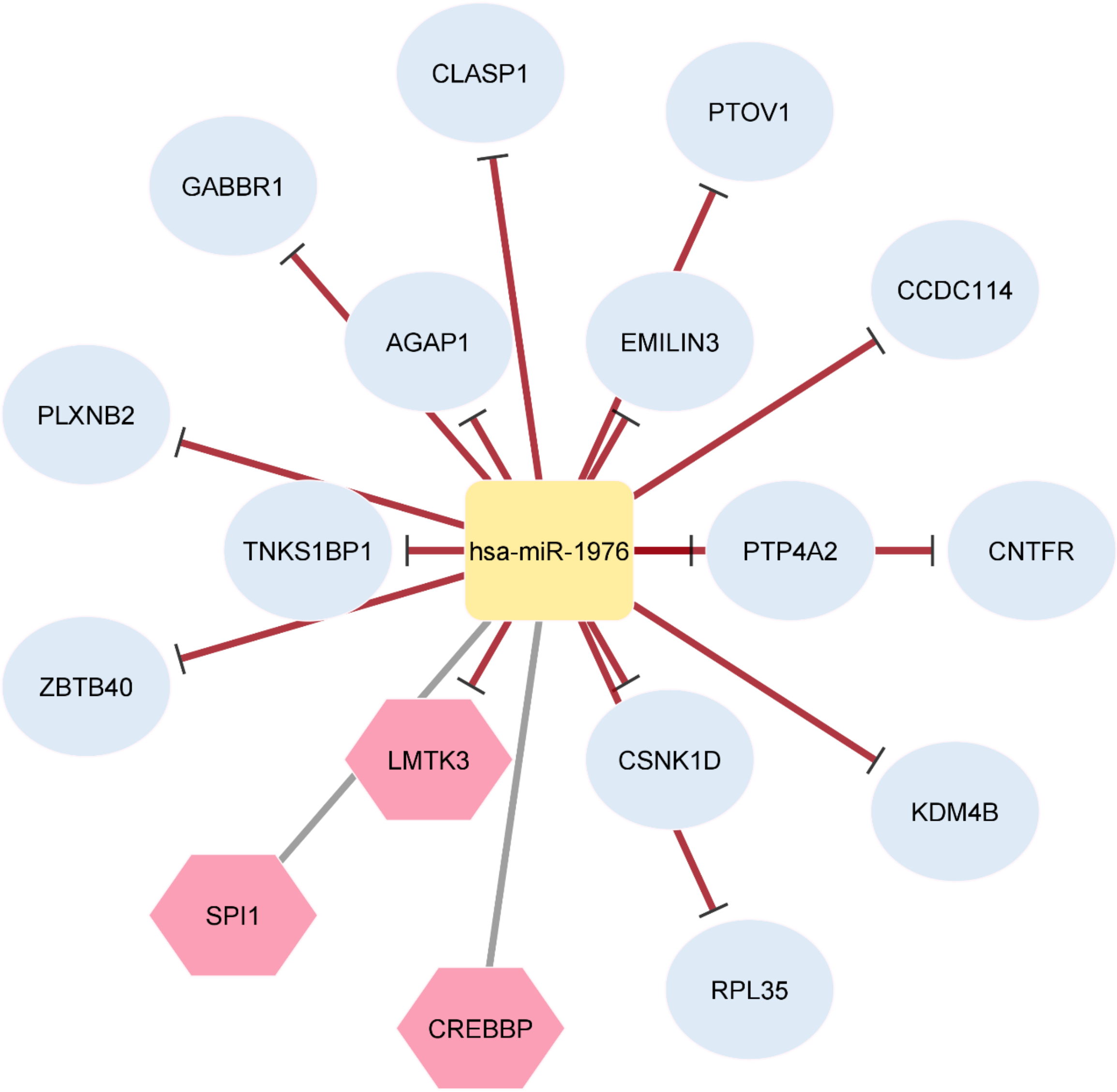

Supplementary Fig. 9 The regulatory network of hsa-miR-1976 includes 18 nodes and 17 edges. Node shape and color are labeled as pink hexagon indicates TFs and yellow round square indicates miRNAs. The nodes have colored and directed edges: T-shaped red edges indicate repression. Black-colored edges indicate regulation.

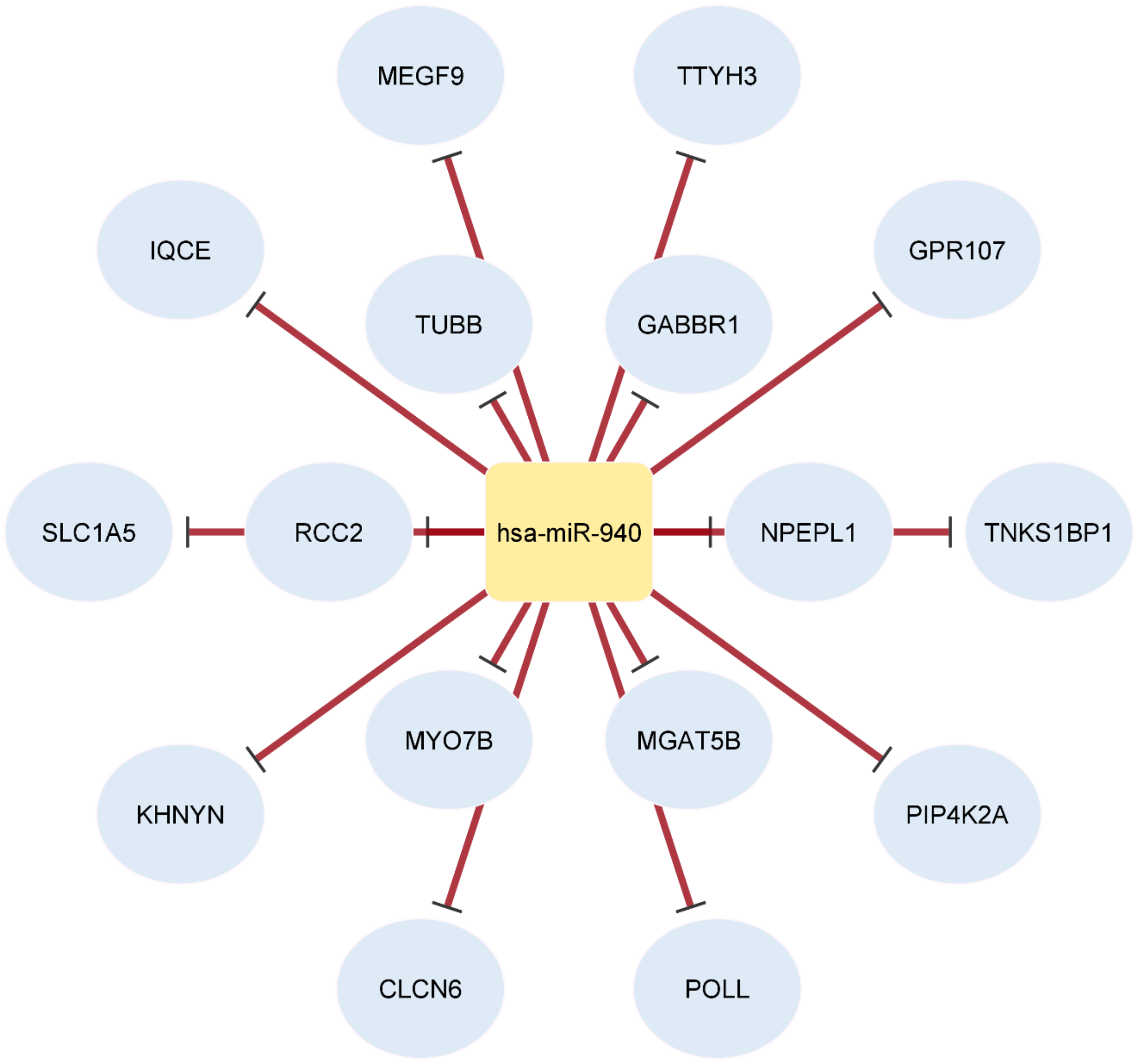

Supplementary Fig. 10 The regulatory network of hsa-miR-940, which contains 17 nodes and 16 edges. Node shape and color are labeled as pink hexagon indicates TFs and yellow round square indicates miRNAs. The nodes have colored and directed edges: T-shaped red edges indicate repression.

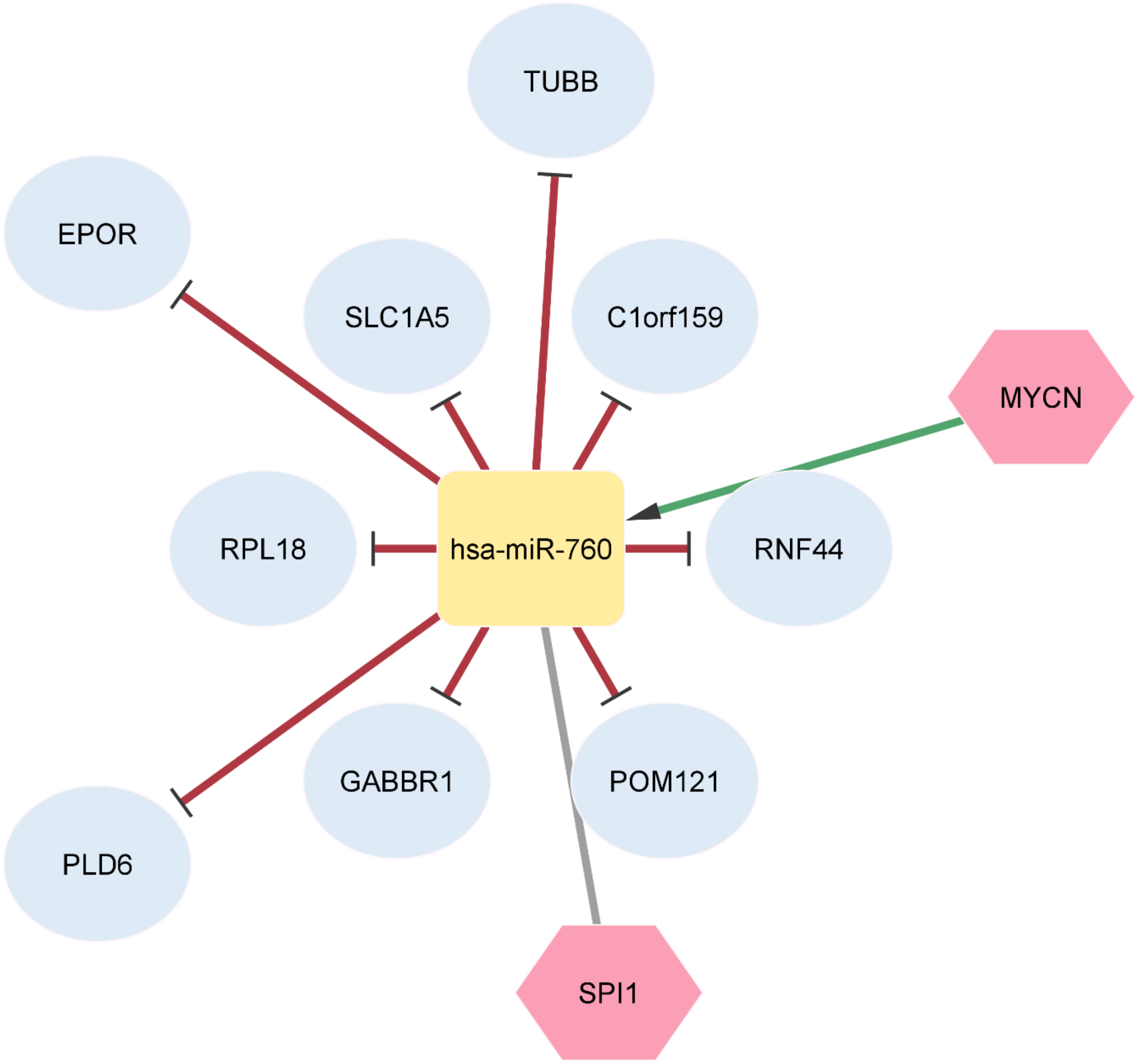

Supplementary Fig. 11 The regulatory network of hsa-miR-760, which contains 12 nodes and 11 edges. Node shape and color are labeled as pink hexagon indicates TFs and yellow round square indicates miRNAs. The nodes have colored and directed edges: T-shaped red edges indicate repression. Delta green arrows indicate activation while black-colored edges indicate regulation.

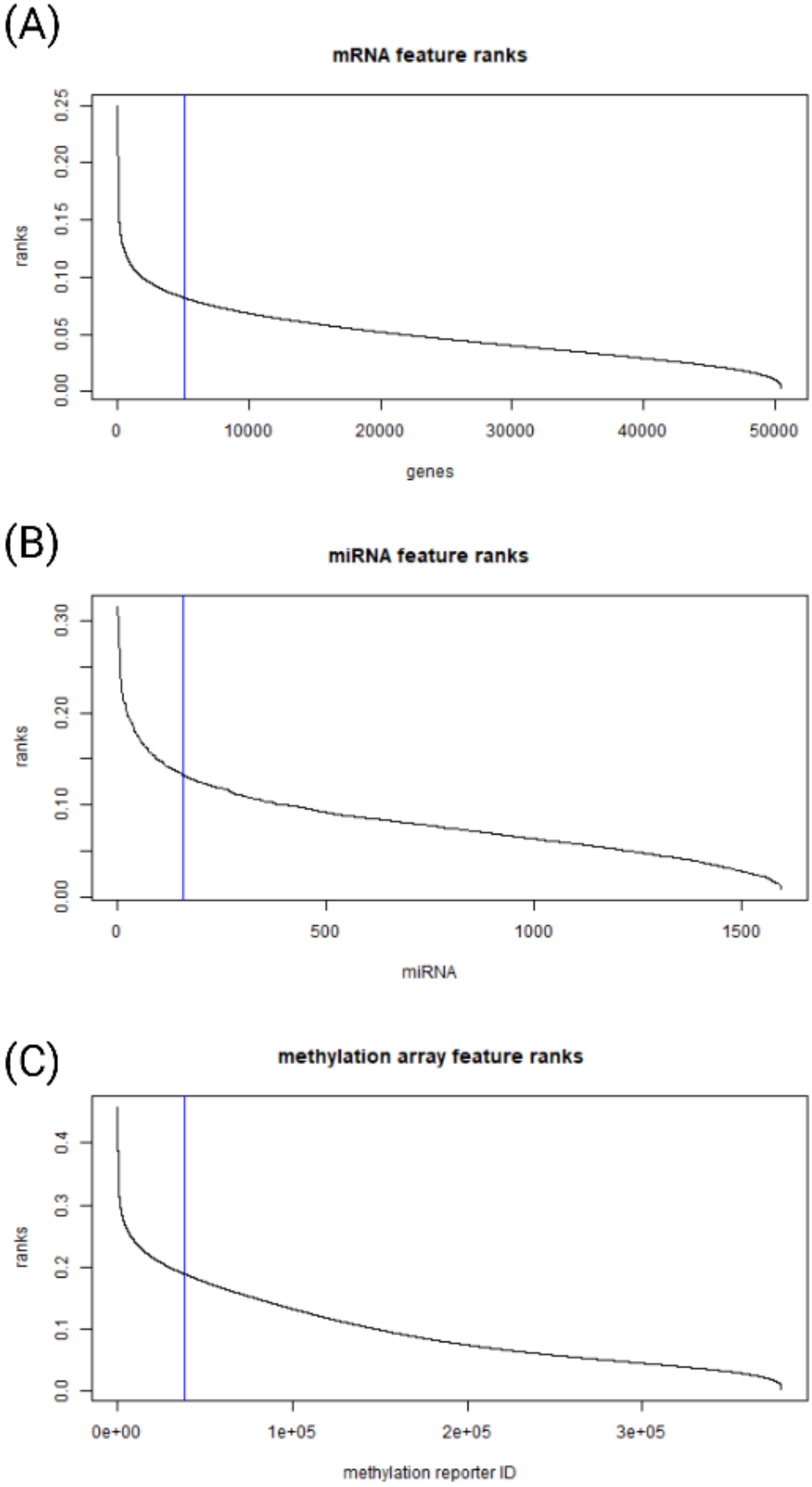
(A)
mRNA feature ranks
ranks
0.00
0.05
0.10
0.15
0.20
0.25
0
10000
20000
30000
40000
50000
genes
(B)
miRNA feature ranks
ranks
0.00
0.10
0.20
0.30
0
500
1000
1500
miRNA
(C)
methylation array feature ranks
ranks
0.0
0.1
0.2
0.3
0.4
0e+00
1e+05
2e+05
3e+05
methylation reporter ID

Supplementary Fig. 12 NMI rank scores for different data types. The x-axis represents the features' IDs. The y-axis represents the NMI scores in descending order to determine the ranks. The blue horizontal line identifies the top 10% high-rank features A) mRNA-seq features. (B) miRNA-seq features (C) Methylation array data features.
